# Supplementary material for: Multi-morbidity and blood pressure trajectories in hypertensive patients: A multiple landmark cohort study
Source: PLoS Med. 2021 Jun 17;18(6):e1003674. doi: 10.1371/journal.pmed.1003674 (PMC8248714; doi:10.1371/journal.pmed.1003674)
Supplement: S1 Fig — (PDF) [file pmed.1003674.s002.pdf]

S1 Fig. Study design schema.

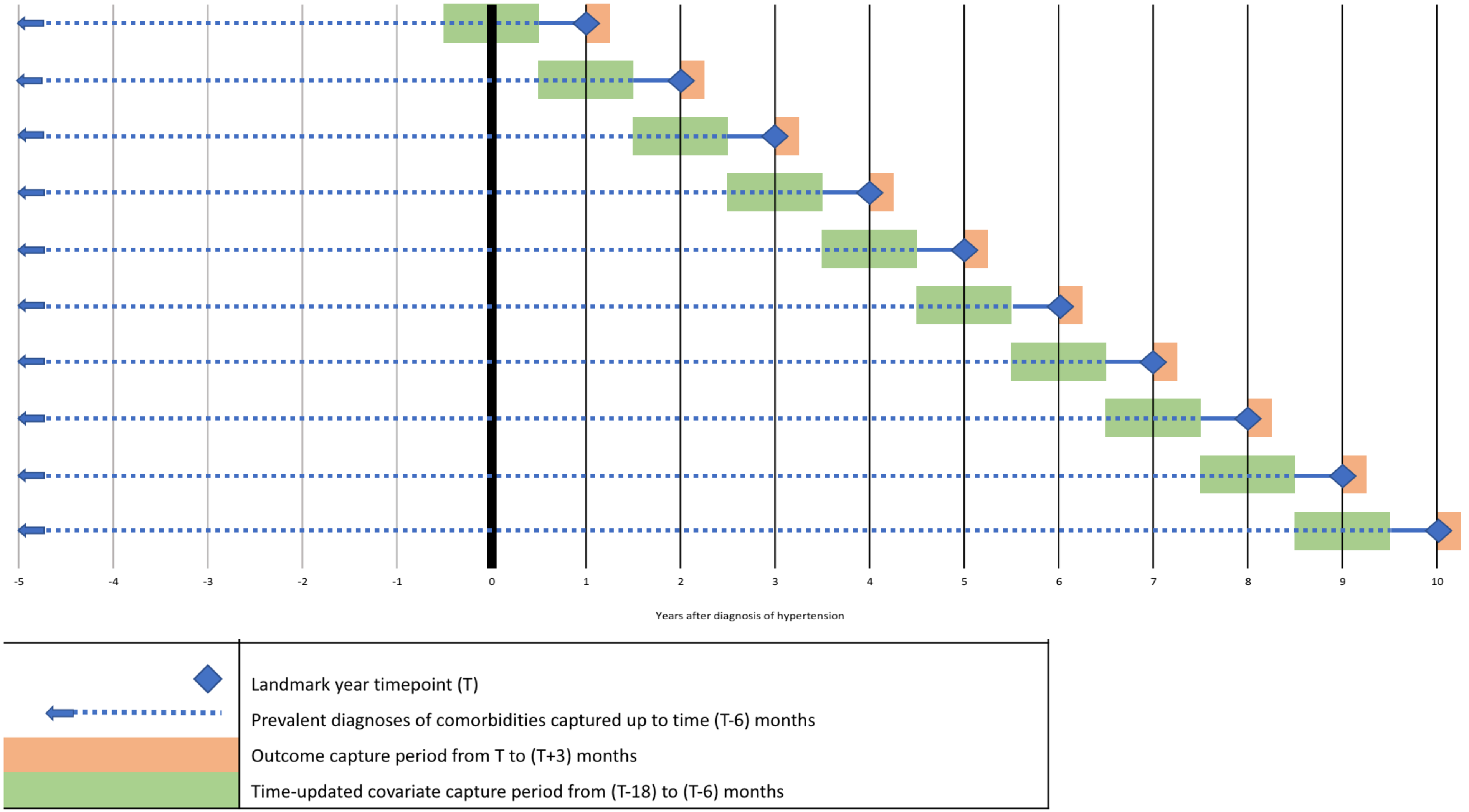

Schematic diagram showing landmark time approach for each cohort analysed. Time-updated covariates were number of prescribed anti-hypertensive classes and cumulative blood pressure. Age was also time-updated at time T-12 months. Covariates that were static or did not have adequate time-varying data, were not updated with each landmark: sex, index of multiple deprivation, gender, ethnicity, body mass index, cholesterol, smoking status and year of hypertension diagnosis; these covariates were measured from up to 12 months before to time of hypertension diagnosis.
