# Supplementary material for: Multi-morbidity and blood pressure trajectories in hypertensive patients: A multiple landmark cohort study
Source: PLoS Med. 2021 Jun 17;18(6):e1003674. doi: 10.1371/journal.pmed.1003674 (PMC8248714; doi:10.1371/journal.pmed.1003674)
Supplement: S2 Fig — (PDF) [file pmed.1003674.s003.pdf]

S2 Fig. Adjusted mean systolic blood pressure over time stratified by number of co-morbidities.

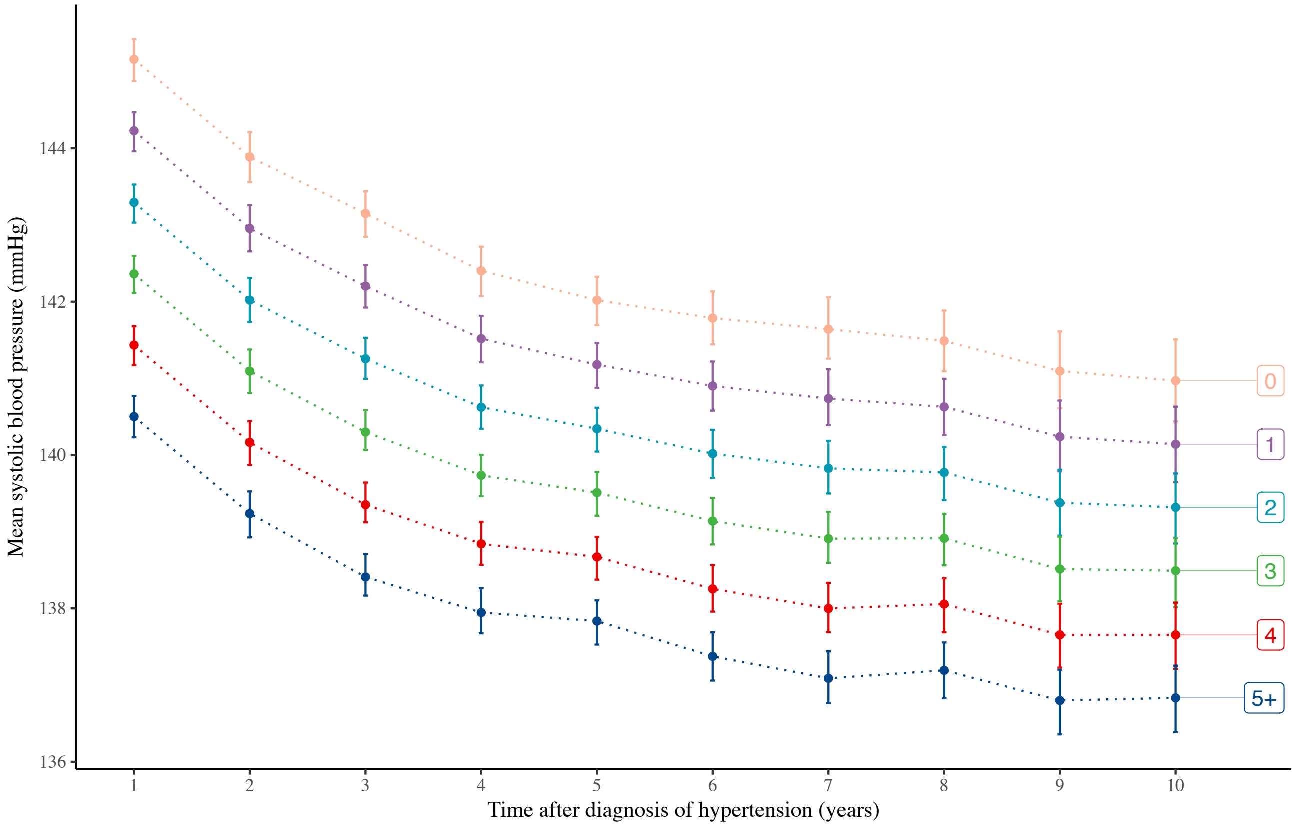

Systolic blood pressure was calculated from linear regression models for each landmark cohort. All models were adjusted for age, sex, index of multiple deprivation, ethnicity, cholesterol, body mass index, smoking status, number of classes of prescribed anti-hypertensive medications and year of diagnosis of hypertension.
