# Supplementary material for: Multi-morbidity and blood pressure trajectories in hypertensive patients: A multiple landmark cohort study
Source: PLoS Med. 2021 Jun 17;18(6):e1003674. doi: 10.1371/journal.pmed.1003674 (PMC8248714; doi:10.1371/journal.pmed.1003674)
Supplement: S3 Fig — (PDF) [file pmed.1003674.s004.pdf]

S3 Fig. Mean systolic blood pressure over time by number of co-morbidities in men.

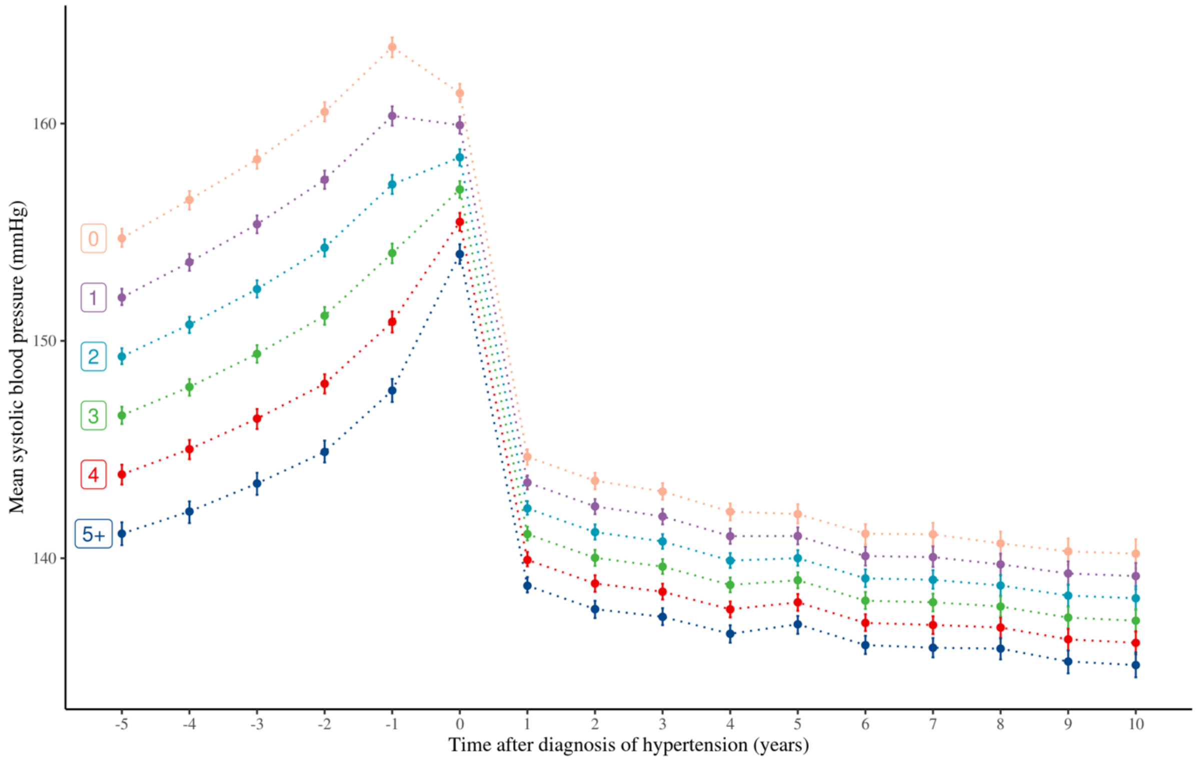

Systolic blood pressure was calculated from linear regression models for each landmark cohort. Each line represents number of co-morbidities in addition to hypertension: 0,1,2,3,4,5+. Bars for each dot represent 95% confidence intervals. Negative time indicates time (year) before diagnosis of hypertension. Models were adjusted for age, sex, deprivation level, ethnicity, body mass index, smoking status, number of classes of prescribed anti-hypertensive medications, and year of diagnosis of hypertension.
