# Supplementary material for: Multi-morbidity and blood pressure trajectories in hypertensive patients: A multiple landmark cohort study
Source: PLoS Med. 2021 Jun 17;18(6):e1003674. doi: 10.1371/journal.pmed.1003674 (PMC8248714; doi:10.1371/journal.pmed.1003674)
Supplement: S4 Fig — (PDF) [file pmed.1003674.s005.pdf]

S4 Fig. Mean systolic blood pressure over time by number of co-morbidities in women.

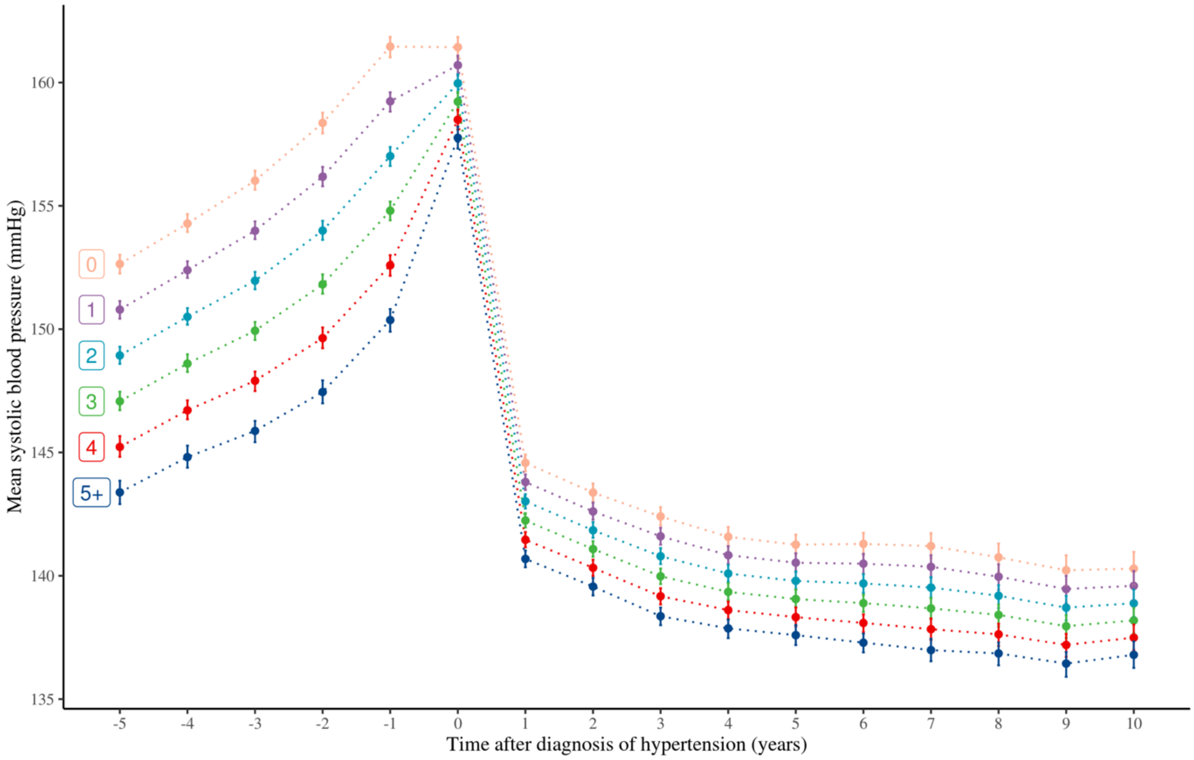

Systolic blood pressure was calculated from linear regression models for each landmark cohort. Each line represents number of co-morbidities in addition to hypertension: 0,1,2,3,4,5+. Bars for each dot represent 95% confidence intervals. Negative time indicates time (year) before diagnosis of hypertension. Models were adjusted for age, sex, deprivation level, ethnicity, body mass index, smoking status, number of classes of prescribed anti-hypertensive medications, and year of diagnosis of hypertension.

Fig S5. Adjusted mean differences in systolic blood pressure at 1 year after hypertension diagnosis in men, stratified by comorbidity status.

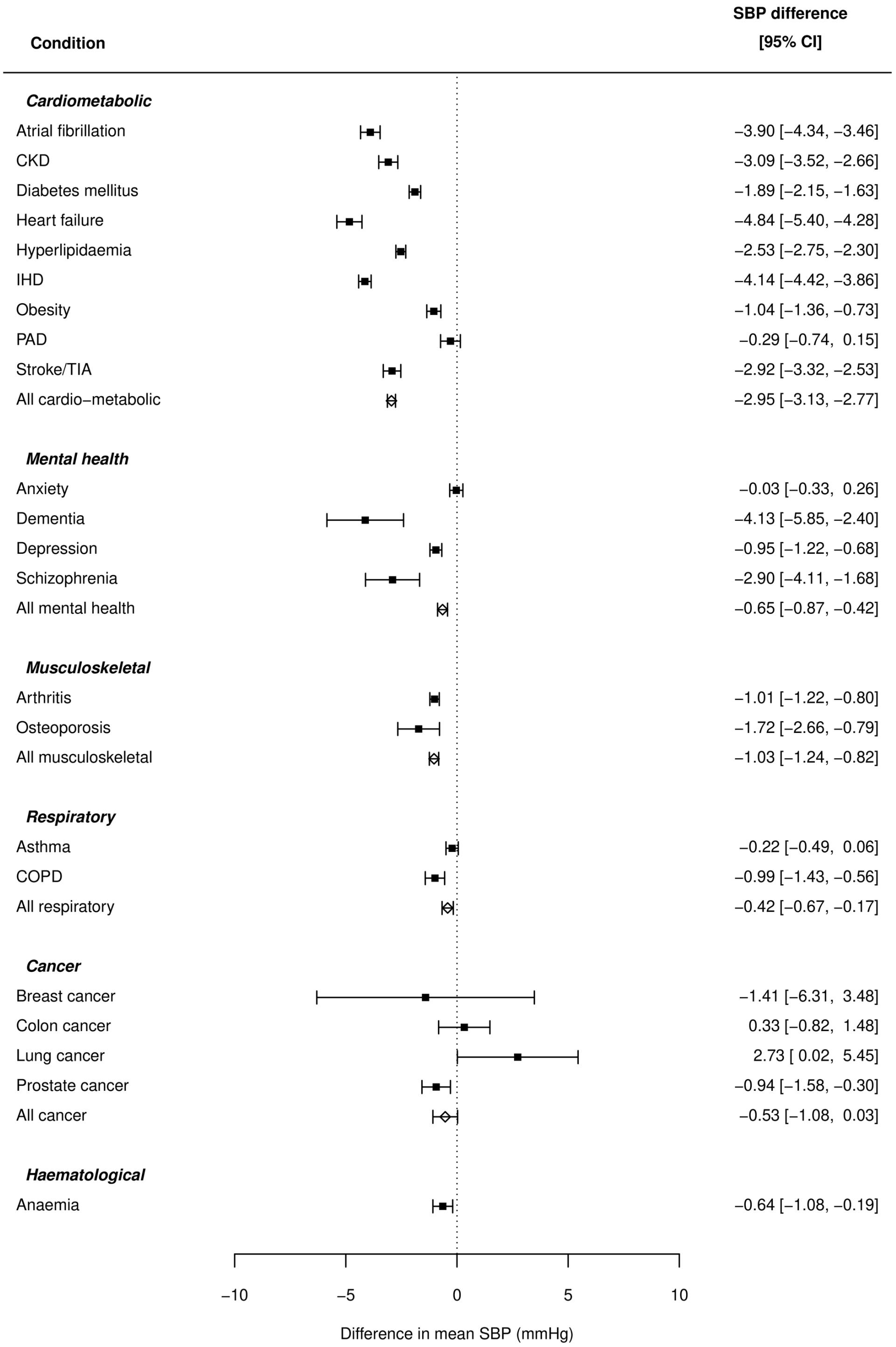

All models were adjusted for age, sex, deprivation level, ethnicity, cholesterol, body mass index, smoking status, number of classes of prescribed anti-hypertensive medications, and year of diagnosis of hypertension. Reference group for each point estimate was patients without that particular co-morbidity. CKD: chronic kidney disease, IHD: ischaemic heart disease, PAD: peripheral arterial disease, TIA: transient ischaemic attack, COPD: chronic obstructive pulmonary disease.

Fig S6. Adjusted mean differences in systolic blood pressure at 1 year after hypertension diagnosis in women, stratified by comorbidity status.

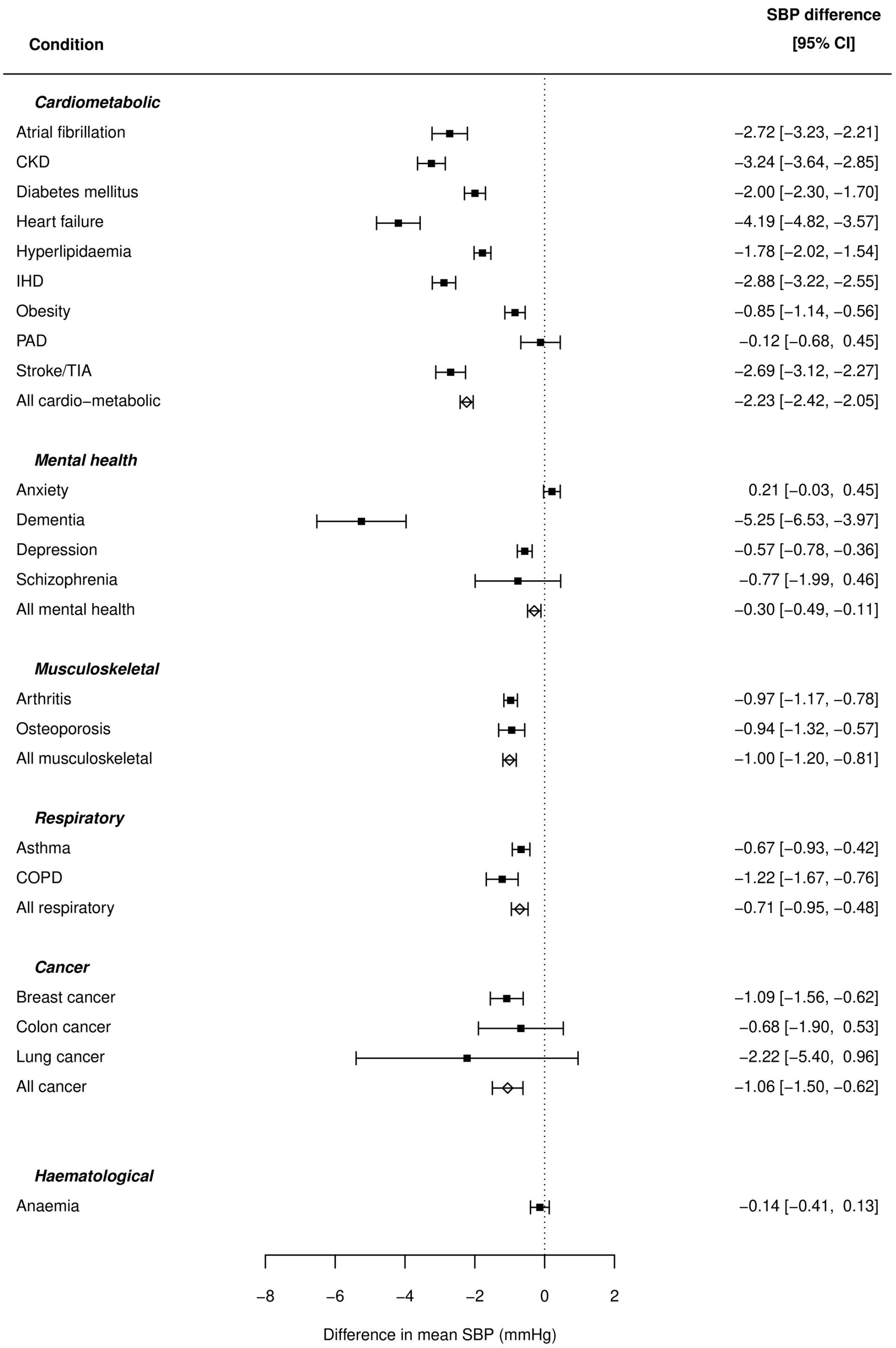

All models were adjusted for age, sex, deprivation level, ethnicity, cholesterol, body mass index, smoking status, number of classes of prescribed anti-hypertensive medications, and year of diagnosis of hypertension. Reference group for each point estimate was patients without that particular co-morbidity. CKD: chronic kidney disease, IHD: ischaemic heart disease, PAD: peripheral arterial disease, TIA: transient ischaemic attack, COPD: chronic obstructive pulmonary disease.

Fig S7. Systolic blood pressure (SBP) stratified by comorbidity – before and at time of hypertension diagnosis.

a) One year before hypertension diagnosis

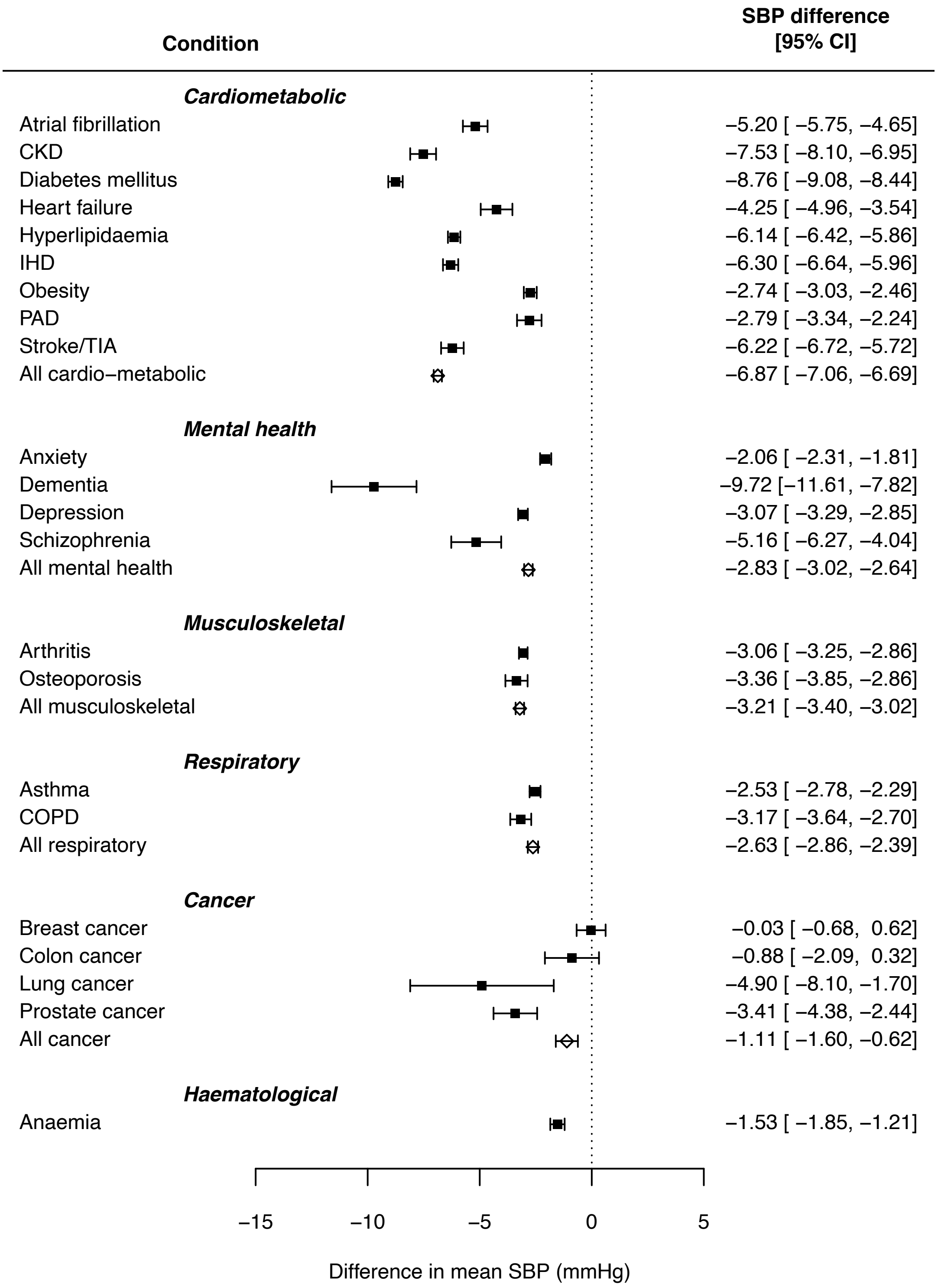

b) At diagnosis

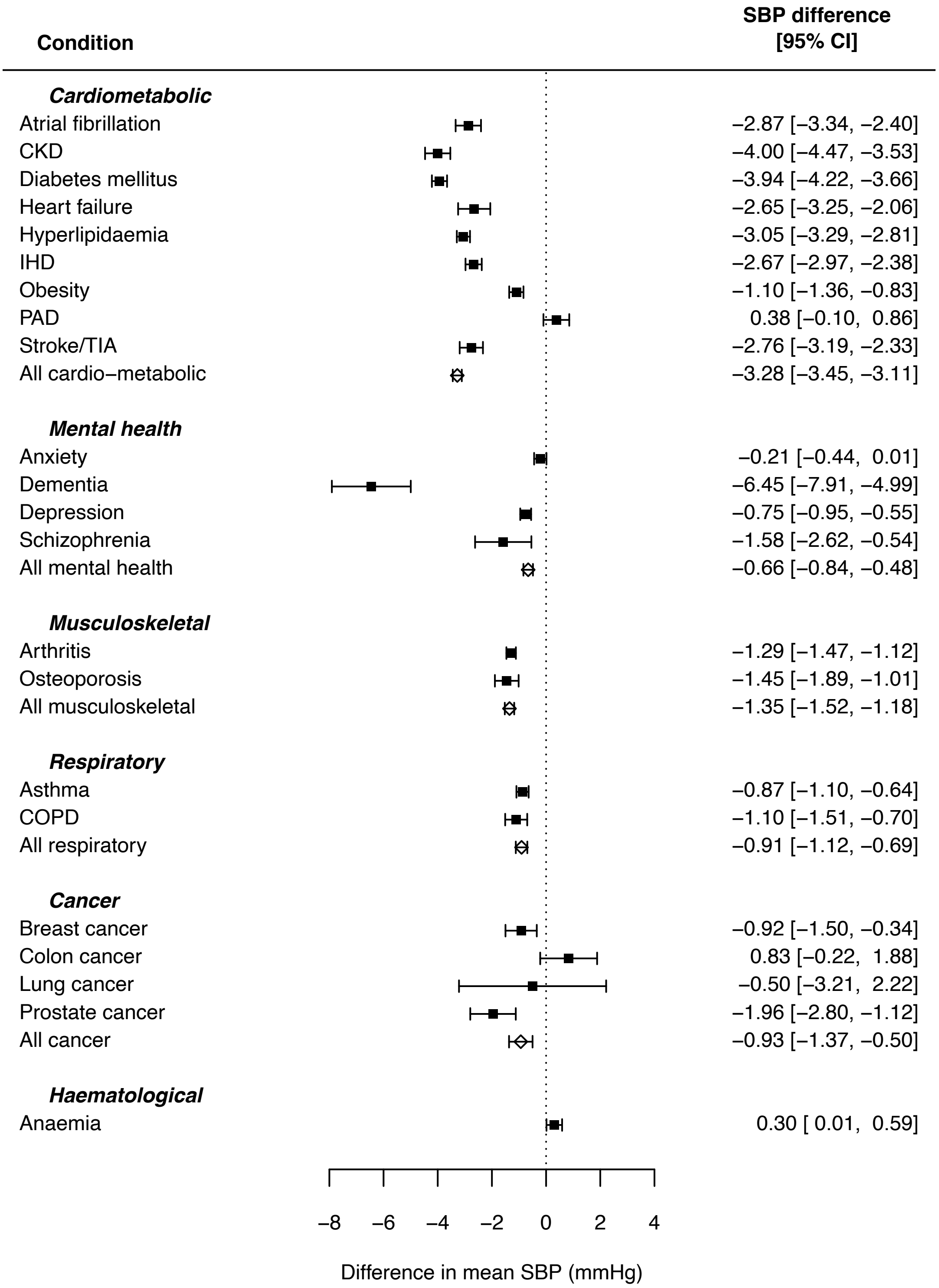

SBP was calculated from simulations using linear regression models on a landmark dataset for each timepoint. All models were adjusted for age, sex, index of multiple deprivation, ethnicity, cholesterol, body mass index, smoking status, number of classes of prescribed anti-hypertensive medications and year of diagnosis of hypertension. Closed circle represents the average SBP of patients with the condition or condition group, open circle represents patients without the condition or condition group, line represents the SBP difference derived from linear regression. PAD: peripheral arterial disease; CKD: chronic kidney disease, IHD: ischaemic heart disease, TIA: transient ischaemic attack, COPD: chronic obstructive pulmonary disease.

Table S1. Diagnostic codes for hypertension.

Medcode	Description
204	Hypertensive disease
799	Essential hypertension
1894	Benign essential hypertension
3712	Hypertension NOS
3979	Hypertensive encephalopathy
4372	Systolic hypertension
4668	Hypertensive renal disease
6702	Hypertensive retinopathy
7057	Hypertensive disease NOS
7329	Secondary hypertension
8732	BP - hypertensive disease
8857	Cardiomegaly - hypertensive
10818	Essential hypertension NOS
15106	Hypertensive renal disease NOS
15377	Malignant essential hypertension
16059	Secondary hypertension NOS
16173	Hypertensive heart disease NOS
16292	Hypertensive heart disease
18057	Antihypertensive therapy
18765	Other specified hypertensive disease
21826	Hypertension treatm. started
21837	Hypertensive heart&renal dis wth (congestive) heart failure
25371	Secondary benign renovascular hypertension
28684	Hypertensive heart and renal disease with renal failure
29310	Renal hypertension
31341	Hypertension secondary to drug
31387	Secondary renovascular hypertension NOS
31464	Hypertensive heart disease NOS
31755	Secondary malignant hypertension
31816	Hypertensive crisis
32423	Hypertensive renal disease with renal failure
34744	Hypertension secondary to endocrine disorders
37086	Blind hypertensive eye
39649	Malignant hypertensive renal disease
42229	Secondary hypertension NOS
43935	Benign hypertensive renal disease
50157	Malignant hypertensive heart disease
51635	Secondary benign hypertension NOS
52127	Benign hypertensive heart disease with CCF
52427	Benign hypertensive heart disease

57288	Secondary benign hypertension
57987	Hyperten heart&renal dis+both(congestv)heart and renal fail
61166	Hypertensive heart disease NOS without CCF
61660	Benign hypertensive heart disease without CCF
62718	Hypertensive heart disease NOS with CCF
63000	Benign hypertensive heart and renal disease
63466	Hypertensive heart and renal disease
67232	Malignant hypertensive heart and renal disease
68659	Hypertensive heart and renal disease NOS
69753	[X]Hypertensive diseases
72668	Malignant hypertensive heart disease with CCF
73293	Secondary malignant hypertension NOS
83473	Diastolic hypertension
95334	Malignant hypertensive heart disease without CCF
97533	[X]Hypertension secondary to other renal disorders

Diagnostic codes for hypertension are the most accurate way of defining hypertensive patients, and this approach has been used extensively in the literature.

Table S2. Comorbidities selected.

Group	Comorbidity	Included conditions
Cardiometabolic	Chronic kidney disease	Chronic kidney disease stage 3 or more, or dependence on transplant or dialysis
	Diabetes mellitus	Includes type I and II, and diabetes-specific sequelae
	Heart failure	
	Hyperlipidaemia	
	Ischaemic heart disease	Includes myocardial infarction, unstable angina, stable angina
	Obesity	
	Peripheral arterial disease	Includes aortic aneurysm and dissection, embolism and thrombosis, and unspecified peripheral vascular diseases
	Stroke/TIA	Includes ischaemic and haemorrhagic stroke, transient ischaemic attack (TIA)
Mental & cognitive health	Anxiety	Anxiety and phobic disorders
	Dementia	Includes Alzheimer disease, vascular dementia, and unspecified dementia
	Depression	Depression and depressive mood disorders
	Schizophrenia	
Respiratory	Asthma	
	Chronic obstructive pulmonary disease	
Musculoskeletal	Arthritis	Chronic arthritis, including osteoarthritis, secondary arthritides (e.g., arthropathy in Crohn's disease and psoriatic arthritis), and other unspecified arthritides and sequelae; excluding acute arthritis (e.g., septic arthritis) and arthritides covered elsewhere: gout, rheumatoid arthritis, and connective-tissue-related arthritis
	Osteoporosis	
Cancers	Breast cancer	Primary cancer of specified organ
	Colon cancer	
	Lung cancer	
	Prostate cancer	
Haematological	Anaemia	

For each co-morbidity, a list of diagnostic codes from hospital (ICD-10) and primary care coding schemes was used to identify diagnoses. The codes were compiled from online code repositories, including Cardiovascular Disease Research Using Linked Bespoke Studies and Electronic Health Records (CALIBER) [1] and medical dictionary keyword searches. These comorbidities were selected based on 3 sources: 1) the Quality and Outcomes Framework, a performance management and incentive scheme for general practitioners in the UK [2], 2) the Charlson Comorbidity Index, a commonly used co-morbidity index [3] and, 3) the US Department of Health and Human Services [4].

Table S3. Anti-hypertensive British National Formulary (BNF) codes and drug classes.

BNF code	Description	Class A	B	C	D	O
2020100	Thiazides and related diuretics				✓	
2020200	Loop diuretics				✓	
2020300	Potassium sparing diuretics & aldosterone antagonists				✓	
2020400	Potassium sparing diuretics & compounds				✓	
2020800	Diuretics with potassium				✓	
2040000	Beta-adrenoceptor blocking drugs		✓			
2040100	Beta-adrenoceptor blocking drugs with diuretic		✓		✓	
2050000	Hypertension and heart failure		✓			
2050100	Vasodilator antihypertensive drugs					✓
2050200	Centrally-acting antihypertensive drugs					✓
2050300	Adrenergic neurone blocking drugs					✓
2050400	Alpha-adrenoceptor blocking drugs					✓
2050500	Renin-angiotensin system drugs					
2050501	Angiotensin-converting enzyme inhibitors	✓				
2050502	Angiotensin-ii receptor antagonists	✓				
2050503	Renin inhibitors					
2050504	Angiotensin-ii receptor antagonists with diuretic	✓			✓	
2050800	Other adrenergic neurone blocking drugs					✓
2060200	Calcium-channel blockers			✓		
2050500*	Renin-angiotensin system drugs					

Classes were defined as: A) Angiontensin-converting enzyme inihibitors and angiotensin II receptor blockers, B) beta-blockers, C) calcium-channel blockers, D) diuretics, O) other (including alpha-blockers, vasodilators, centrally-acting anti-hypertensives); *Each drug under this code was individually categorised into a class.

Table S4. Descriptive statistics of blood pressure management before and after diagnosis of hypertension.

	Mean number of BP readings (SD)*		Mean number of antihypertensive classes prescribed (SD)#	
**Number of comorbidities**	**Before diagnosis**	**After diagnosis**	**Before diagnosis**	**After diagnosis**
**Total**	8.0 (6.6)	15.5 (10.0)	0.3 (0.7)	1.5 (0.9)
**0**	6.3 (5.4)	15.1 (9.7)	0.1 (0.5)	1.5 (0.9)
**1**	7.7 (6.1)	15.6 (10.0)	0.2 (0.6)	1.5 (0.8)
**2**	9.0 (6.8)	15.7 (10.2)	0.3 (0.7)	1.5 (0.9)
**3**	10.2 (7.4)	15.9 (10.6)	0.5 (0.8)	1.6 (0.9)
**4**	11.3 (7.9)	16.0 (11.0)	0.6 (0.9)	1.7 (0.9)
**5+**	13.7 (9.0)	16.0 (11.8)	1.0 (1.1)	1.9 (1.0)

*Capture period was 5 years from diagnosis of hypertension i.e. mean number of blood pressure (BP) readings were counted from 5 years before diagnosis to time of diagnosis, and from time of diagnosis to 5 years after diagnosis. #Capture period was 1 year from time of diagnosis of hypertension i.e. mean number of anti-hypertensive classes prescribed were counted from 1 year before diagnosis to time of diagnosis, and from time of diagnosis to 1 year after diagnosis. All differences between groups statistically significant (p<0.01) using analysis of variance

Table S5. Patient characteristics in landmark cohorts of incident hypertensive patients between 2000 and 2014 in the UK.

Characteristic	Time after diagnosis of hypertension (years)					
	-5 (n=145,964)	-1 (n=273,493)	0 (n=295,487)	1 (n=260,823)	5 (n=158,573)	10 (n=52,393)
**Age [years], mean (SD)**	56.6 (12.7)	60.5 (13.1)	61.5 (13.1)	62.6 (12.9)	66.2 (12.4)	70 (11.5)
**<65, % (n)**	74.4 (108,551)	63.7 (174,146)	60.7 (179,239)	57.8 (150,784)	47.6 (75,421)	35.2 (18,424)
**≥65, % (n)**	25.6 (37,413)	36.3 (99,347)	39.3 (116,248)	42.2 (110,039)	52.4 (83,152)	64.8 (33,969)
**Women, % (n)**	49.0 (71,482)	50.3 (137,700)	50.7 (149,787)	51.0 (133,098)	51.9 (82,375)	53.9 (28,247)
**Number of comorbidities**						
**0**	47.9 (69,856)	39.3 (107,551)	36.1 (106,801)	27.6 (72,090)	17.6 (27,979)	10.7 (5,594)
**1**	29.3 (42,719)	29.5 (80,549)	29.3 (86,691)	29.7 (77,399)	25.2 (39,945)	19.3 (10,107)
**2**	14.0 (20,397)	16.8 (46,059)	17.7 (52,432)	20.6 (53,744)	22.1 (35,100)	21.1 (11,066)
**3**	5.7 (8,272)	8.2 (22,365)	9.2 (27,185)	11.6 (30,358)	15.5 (24,653)	17.6 (9,195)
**4**	2.1 (3,119)	3.7 (10,090)	4.3 (12,824)	5.9 (15,287)	9.4 (14,901)	12.7 (6,663)
**≥5**	1.1 (1,601)	2.5 (6,879)	3.2 (9,554)	4.6 (11,945)	10.1 (15,995)	18.6 (9,768)
**No. with blood pressure measurements, % with missing data [n]**	36,350<br>87.7 [259,137]	69,622<br>76.4 [225,865]	271,002<br>8.3 [24,485]	142,778<br>46.9 [126,051]	80,446<br>52.0 [87,144]	28,049<br>51.7 [30,052]

Each timepoint represents a landmark year cohort (see Methods)

Table S6. Baseline characteristics by cardiometabolic co-morbidity.

Characteristic	All cases (n=295,487)	Cardiometabolic comorbidities	
		Yes (n=112,656)	No (n=182,831)
**Age [years], mean (SD)**	61.5 (13.1)	63.5 (13.0)	60.3 (13.0)
**<65, % (n)**	60.7 (179,239)	54.0 (60,780)	64.8 (118,459)
**≥65, % (n)**	39.3 (116,248)	46.0 (51,876)	35.2 (64,372)
**Women, % (n)**	50.7 (149,787)	48.3 (54,437)	52.2 (95,350)
**White ethnicity, % (n)**	96.1 (116,574)	95.8 (47,723)	96.3 (68,851)
	[174,197]	[62,848]	[111,349]
**Fifths of deprivation index, % (n)**			
**Q1 (least deprived)**	23.3 (68,902)	21.3 (23,961)	24.6 (44,941)
**Q2**	22.6 (66,699)	21.4 (24,080)	23.3 (42,619)
**Q3**	21.0 (62,142)	21.0 (23,663)	21.0 (38,479)
**Q4**	18.2 (53,722)	19.2 (21,610)	17.6 (32,112)
**Q5 (most deprived)**	14.8 (43,718)	17.1 (19,220)	13.4 (24,498)
**Mean SBP (SD), mmHg**	159.1 (21.1)	155.8 (20.7)	161.1 (21.1)
**Mean DBP (SD), mmHg**	91.0 (12.5)	88.0 (12.5)	92.9 (12.1)
**Body mass index kg/m^2, % (n)**			
**Underweight**	0.9 (1,552)	0.7 (581)	1.0 (971)
**Normal**	22.0 (37,849)	18.1 (14,156)	25.2 (23,693)
**Overweight**	38.6 (66,484)	35.1 (27,482)	41.5 (39,002)
**Obese**	38.5 (66,403)	46.1 (36,117)	32.2 (30,286)
	[123,199]	[34,320]	[88,879]
**Smoking status, % (n)**			
**Current**	20.1 (44,376)	18.9 (16,991)	21.0 (27,385)
**Never**	47.7 (105,304)	44.7 (40,195)	49.9 (65,109)
**Ex-smoker**	32.1 (70,871)	36.5 (32,809)	29.2 (38,062)
	[74,936]	[22,661]	[52,275]
**Mean total cholesterol (SD), mmol/L**	5.5 (1.2)	5.3 (1.3)	5.7 (1.0)
	[104,581]	[23,989]	[80,592]
**Year of hypertension diagnosis, % (n)**			
**2000**	5.0 (14,822)	4.4 (4,918)	5.4 (9.904)
**2014**	3.4 (10,129)	4.0 (4,477)	3.1 (5,652)
**Number of anti-hypertensive classes prescribed, % (n)**			
**1**	13.9 (41,137)	18.7 (21,113)	11.0 (20,024)
**2**	6.5 (19,267)	10.4 (11,670)	4.2 (7,597)
**≥3**	2.7 (8,058)	5.0 (5,620)	1.3 (2,438)

The category percentages refer to complete cases. Numbers in square brackets are numbers with missing data for the relevant characteristic. Deprivation level refers to Index of Multiple Deprivation (IMD) 2015, where Q1 is least deprived fifth and Q5 is most deprived fifth of the population. SBP – systolic blood pressure; DBP – diastolic blood pressure. BMI categories are "underweight" (<18.5 kg/m^2), "normal" (18.5-24.9 kg/m^2), "overweight" (25-29.9 kg/m^2), and "obese" (>=30 kg/m^2). Number of antihypertensive classes refers to anti-hypertensives grouped into 5 classes: angiotensin-converting enzyme inhibitor and angiotension II receptor blocker, beta-blockers, calcium channel blockers, diuretics, and other (see Methods and Table S1).

References

1. Denaxas SC, George J, Herrett E, Shah AD, Kalra D, Hingorani AD, et al. Data resource profile: cardiovascular disease research using linked bespoke studies and electronic health records (CALIBER). Int J Epidemiol. 2012;41(6):1625-38. Epub 2012/12/12. doi: 10.1093/ije/dys188. PubMed PMID: 23220717; PubMed Central PMCID: PMCPMC3535749.
2. NHS Digital. Quality and Outcomes Framework: NHS Digital; [26 Jul 2020]. Available from: https://qof.digital.nhs.uk/.
3. Charlson ME, Pompei P, Ales KL, MacKenzie CR. A new method of classifying prognostic comorbidity in longitudinal studies: development and validation. J Chronic Dis. 1987;40(5):373-83. Epub 1987/01/01. doi: 10.1016/0021-9681(87)90171-8. PubMed PMID: 3558716.
4. US Department of Health and Human Services Office of the Assistant Secretary for Health. HHS Initiative on Multiple Chronic Conditions 2015 [26 Jul 2020]. Available from: https://www.hhs.gov/ash/about-ash/multiple-chronic-conditions/index.html.
