# Supplementary material for: Multi-morbidity and blood pressure trajectories in hypertensive patients: A multiple landmark cohort study
Source: PLoS Med. 2021 Jun 17;18(6):e1003674. doi: 10.1371/journal.pmed.1003674 (PMC8248714; doi:10.1371/journal.pmed.1003674)
Supplement: S5 Fig — (PDF) [file pmed.1003674.s006.pdf]

S5 Fig. Adjusted mean differences in systolic blood pressure at 1 year after hypertension diagnosis in men, stratified by comorbidity status.

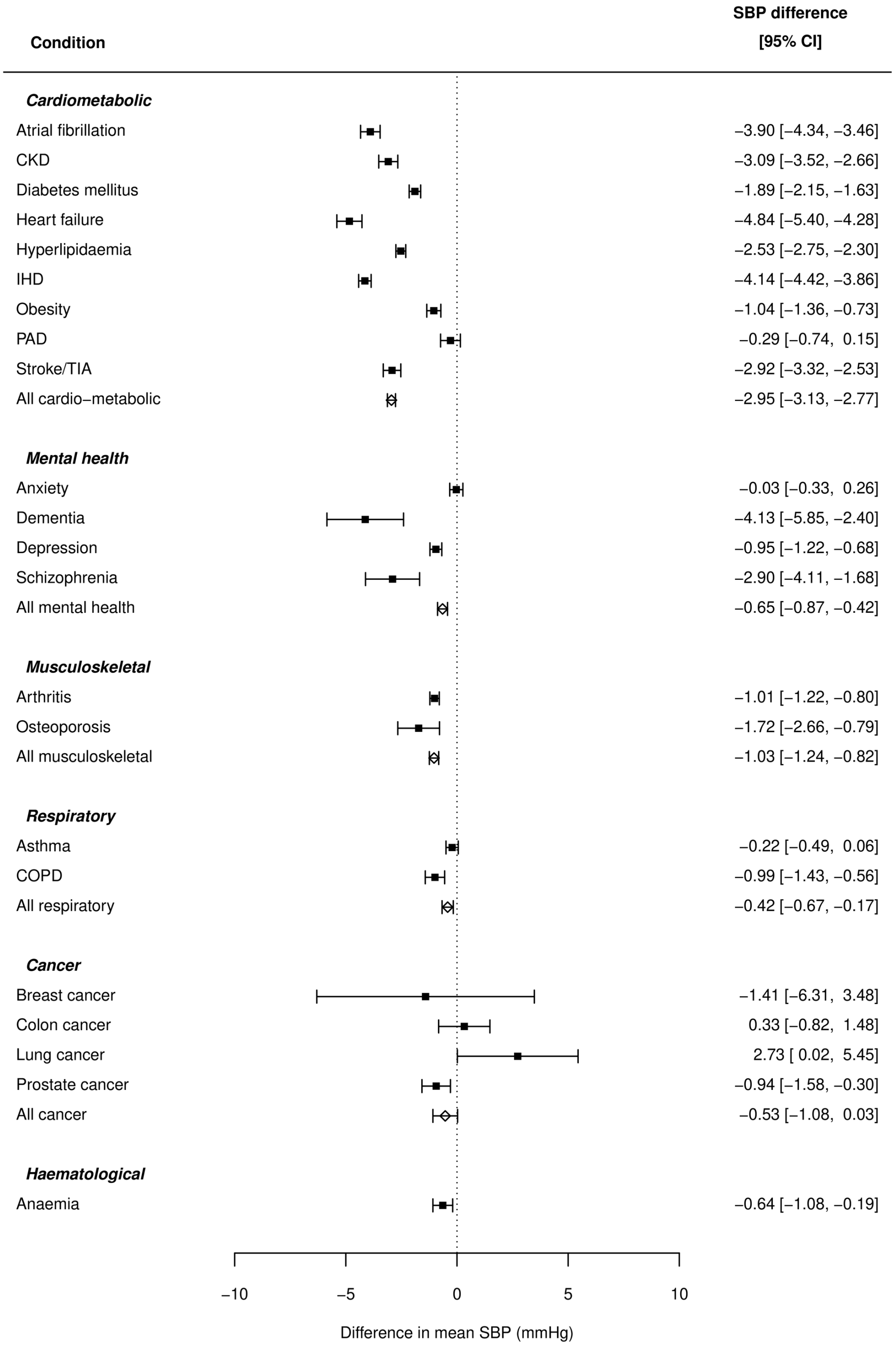

All models were adjusted for age, sex, deprivation level, ethnicity, cholesterol, body mass index, smoking status, number of classes of prescribed anti-hypertensive medications, and year of diagnosis of hypertension. Reference group for each point estimate was patients without that particular co-morbidity. CKD: chronic kidney disease, IHD: ischaemic heart disease, PAD: peripheral arterial disease, TIA: transient ischaemic attack, COPD: chronic obstructive pulmonary disease.
