# Supplementary material for: Multi-morbidity and blood pressure trajectories in hypertensive patients: A multiple landmark cohort study
Source: PLoS Med. 2021 Jun 17;18(6):e1003674. doi: 10.1371/journal.pmed.1003674 (PMC8248714; doi:10.1371/journal.pmed.1003674)
Supplement: S6 Fig — (PDF) [file pmed.1003674.s007.pdf]

S6 Fig. Adjusted mean differences in systolic blood pressure at 1 year after hypertension diagnosis in women, stratified by co-morbidity status.

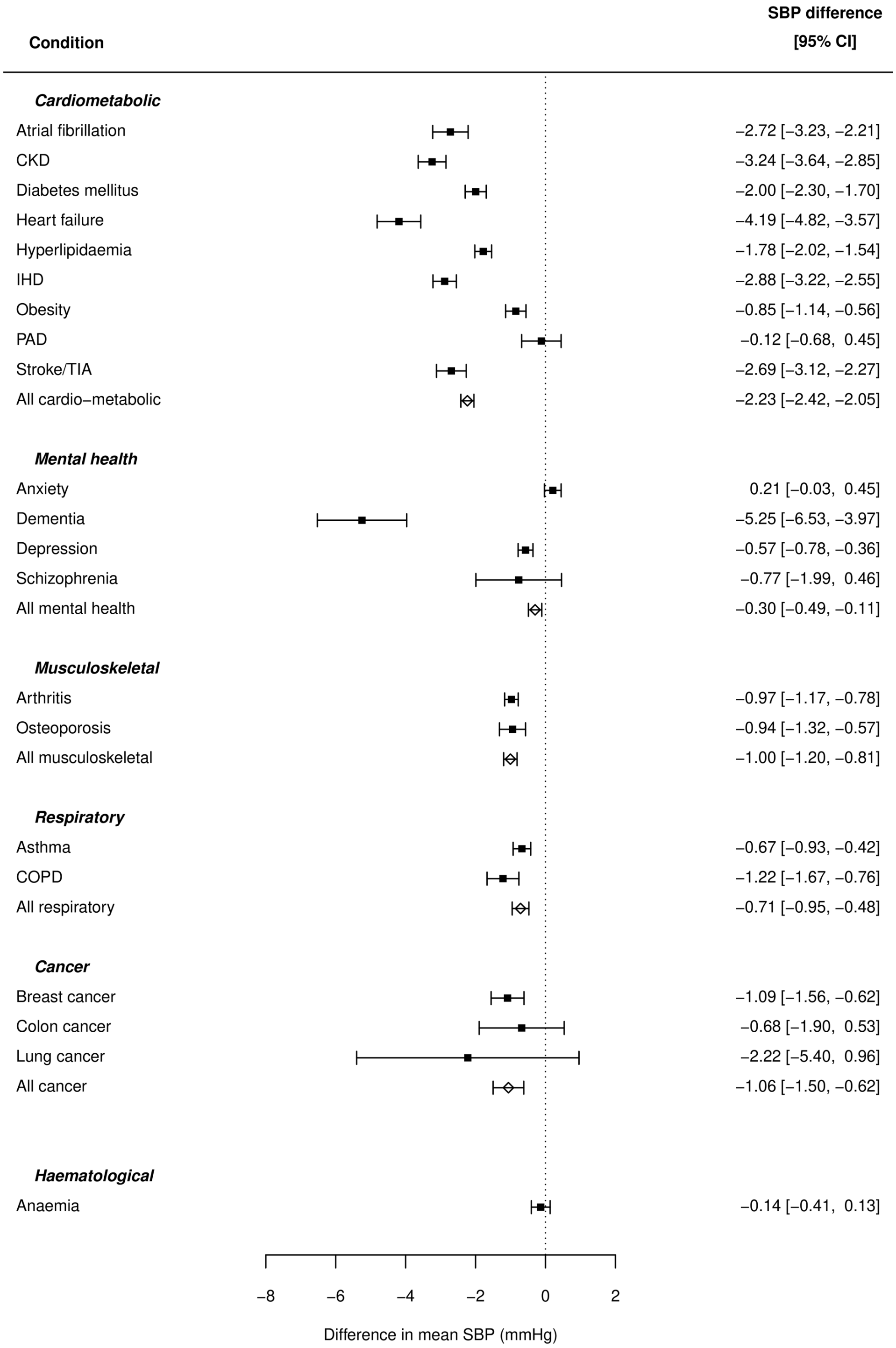

All models were adjusted for age, sex, deprivation level, ethnicity, cholesterol, body mass index, smoking status, number of classes of prescribed anti-hypertensive medications, and year of diagnosis of hypertension. Reference group for each point estimate was patients without that particular co-morbidity. CKD: chronic kidney disease, IHD: ischaemic heart disease, PAD: peripheral arterial disease, TIA: transient ischaemic attack, COPD: chronic obstructive pulmonary disease.
