# Supplementary material for: Multi-morbidity and blood pressure trajectories in hypertensive patients: A multiple landmark cohort study
Source: PLoS Med. 2021 Jun 17;18(6):e1003674. doi: 10.1371/journal.pmed.1003674 (PMC8248714; doi:10.1371/journal.pmed.1003674)
Supplement: S7 Fig — (PDF) [file pmed.1003674.s008.pdf]

S7 Fig. Systolic blood pressure (SBP) before and at time of hypertension diagnosis, stratified by co-morbidities.

a) One year before hypertension diagnosis

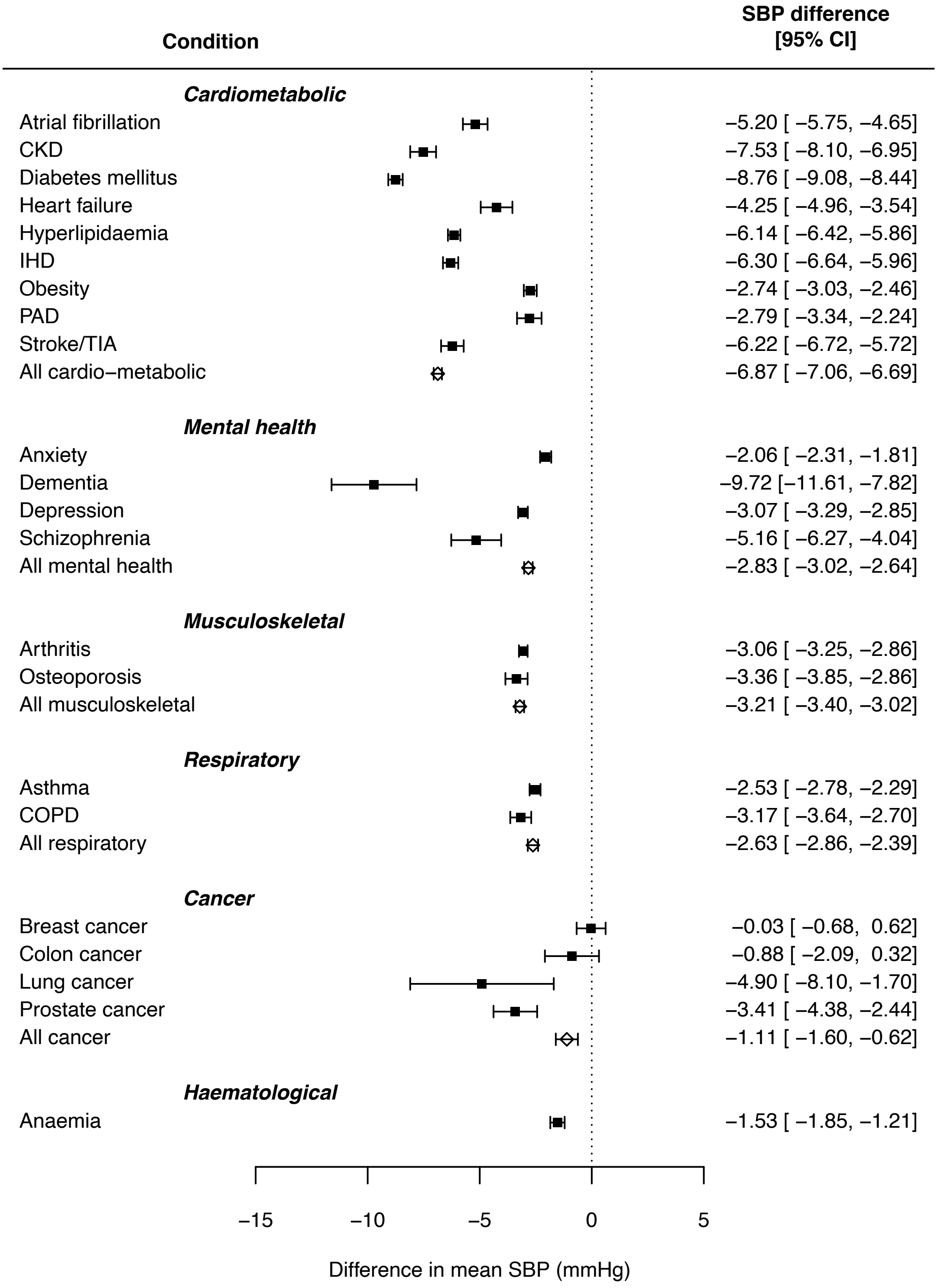

b) At diagnosis

Condition	SBP difference [95% CI]
Cardiometabolic	
Atrial fibrillation	−2.87 [−3.34, −2.40]
CKD	−4.00 [−4.47, −3.53]
Diabetes mellitus	−3.94 [−4.22, −3.66]
Heart failure	−2.65 [−3.25, −2.06]
Hyperlipidaemia	−3.05 [−3.29, −2.81]
IHD	−2.67 [−2.97, −2.38]
Obesity	−1.10 [−1.36, −0.83]
PAD	0.38 [−0.10, 0.86]
Stroke/TIA	−2.76 [−3.19, −2.33]
All cardio−metabolic	−3.28 [−3.45, −3.11]
Mental health	
Anxiety	−0.21 [−0.44, 0.01]
Dementia	−6.45 [−7.91, −4.99]
Depression	−0.75 [−0.95, −0.55]
Schizophrenia	−1.58 [−2.62, −0.54]
All mental health	−0.66 [−0.84, −0.48]
Musculoskeletal	
Arthritis	−1.29 [−1.47, −1.12]
Osteoporosis	−1.45 [−1.89, −1.01]
All musculoskeletal	−1.35 [−1.52, −1.18]
Respiratory	
Asthma	−0.87 [−1.10, −0.64]
COPD	−1.10 [−1.51, −0.70]
All respiratory	−0.91 [−1.12, −0.69]
Cancer	
Breast cancer	−0.92 [−1.50, −0.34]
Colon cancer	0.83 [−0.22, 1.88]
Lung cancer	−0.50 [−3.21, 2.22]
Prostate cancer	−1.96 [−2.80, −1.12]
All cancer	−0.93 [−1.37, −0.50]
Haematological	
Anaemia	0.30 [0.01, 0.59]

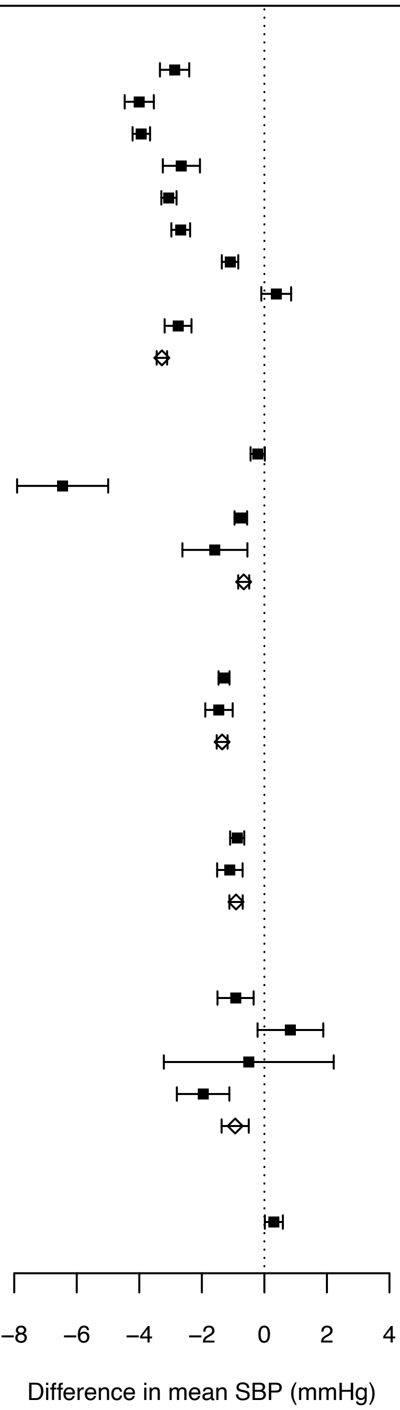

SBP was calculated from simulations using linear regression models on a landmark dataset for each timepoint. All models were adjusted for age, sex, index of multiple deprivation, ethnicity, cholesterol, body mass index, smoking status, number of classes of prescribed anti-hypertensive medications and year of diagnosis of hypertension. Closed circle represents the average SBP of patients with the condition or condition group, open circle represents patients without the condition or condition group, line represents the SBP difference derived from linear regression. PAD: peripheral arterial disease; CKD: chronic kidney disease, IHD: ischaemic heart disease, TIA: transient ischaemic attack, COPD: chronic obstructive pulmonary disease.
