# Supplementary material for: Multi-morbidity and blood pressure trajectories in hypertensive patients: A multiple landmark cohort study
Source: PLoS Med. 2021 Jun 17;18(6):e1003674. doi: 10.1371/journal.pmed.1003674 (PMC8248714; doi:10.1371/journal.pmed.1003674)
Supplement: S1 Table — (PDF) [file pmed.1003674.s009.pdf]

S1 Table. Diagnostic codes for hypertension.

Medcode	Description
204	Hypertensive disease
799	Essential hypertension
1894	Benign essential hypertension
3712	Hypertension NOS
3979	Hypertensive encephalopathy
4372	Systolic hypertension
4668	Hypertensive renal disease
6702	Hypertensive retinopathy
7057	Hypertensive disease NOS
7329	Secondary hypertension
8732	BP - hypertensive disease
8857	Cardiomegaly - hypertensive
10818	Essential hypertension NOS
15106	Hypertensive renal disease NOS
15377	Malignant essential hypertension
16059	Secondary hypertension NOS
16173	Hypertensive heart disease NOS
16292	Hypertensive heart disease
18057	Antihypertensive therapy
18765	Other specified hypertensive disease
21826	Hypertension treatm. started
21837	Hypertensive heart&renal dis wth (congestive) heart failure
25371	Secondary benign renovascular hypertension
28684	Hypertensive heart and renal disease with renal failure
29310	Renal hypertension
31341	Hypertension secondary to drug
31387	Secondary renovascular hypertension NOS
31464	Hypertensive heart disease NOS
31755	Secondary malignant hypertension
31816	Hypertensive crisis
32423	Hypertensive renal disease with renal failure
34744	Hypertension secondary to endocrine disorders
37086	Blind hypertensive eye
39649	Malignant hypertensive renal disease
42229	Secondary hypertension NOS
43935	Benign hypertensive renal disease
50157	Malignant hypertensive heart disease
51635	Secondary benign hypertension NOS
52127	Benign hypertensive heart disease with CCF

52427	Benign hypertensive heart disease
57288	Secondary benign hypertension
57987	Hyperten heart&renal dis+both(congestv)heart and renal fail
61166	Hypertensive heart disease NOS without CCF
61660	Benign hypertensive heart disease without CCF
62718	Hypertensive heart disease NOS with CCF
63000	Benign hypertensive heart and renal disease
63466	Hypertensive heart and renal disease
67232	Malignant hypertensive heart and renal disease
68659	Hypertensive heart and renal disease NOS
69753	[X]Hypertensive diseases
72668	Malignant hypertensive heart disease with CCF
73293	Secondary malignant hypertension NOS
83473	Diastolic hypertension
95334	Malignant hypertensive heart disease without CCF
97533	[X]Hypertension secondary to other renal disorders

Diagnostic codes for hypertension are the most accurate way of defining hypertensive patients, and this approach has been used extensively in the literature.
