# Supplementary material for: Multi-morbidity and blood pressure trajectories in hypertensive patients: A multiple landmark cohort study
Source: PLoS Med. 2021 Jun 17;18(6):e1003674. doi: 10.1371/journal.pmed.1003674 (PMC8248714; doi:10.1371/journal.pmed.1003674)
Supplement: S2 Table — (PDF) [file pmed.1003674.s010.pdf]

S2 Table. Co-morbidities selected in the study.

Group	Co-morbidity	Included conditions
Cardiometabolic	Chronic kidney disease	Chronic kidney disease stage 3 or more, or dependence on transplant or dialysis
	Diabetes mellitus	Includes type I and II, and diabetes-specific sequelae
	Heart failure	
	Hyperlipidaemia	
	Ischaemic heart disease	Includes myocardial infarction, unstable angina, stable angina
	Obesity	
	Peripheral arterial disease	Includes aortic aneurysm and dissection, embolism and thrombosis, and unspecified peripheral vascular diseases
	Stroke/TIA	Includes ischaemic and haemorrhagic stroke, transient ischaemic attack (TIA)
Mental & cognitive health	Anxiety	Anxiety and phobic disorders
	Dementia	Includes Alzheimer disease, vascular dementia, and unspecified dementia
	Depression	Depression and depressive mood disorders
	Schizophrenia	
Respiratory	Asthma	
	Chronic obstructive pulmonary disease	
Musculoskeletal	Arthritis	Chronic arthritis, including osteoarthritis, secondary arthritides (e.g., arthropathy in Crohn's disease and psoriatic arthritis), and other unspecified arthritides and sequelae; excluding acute arthritis (e.g., septic arthritis) and arthritides covered elsewhere: gout, rheumatoid arthritis, and connective-tissue-related arthritis
	Osteoporosis	
Cancers	Breast cancer	Primary cancer of specified organ
	Colon cancer	
	Lung cancer	
	Prostate cancer	
Haematological	Anaemia	

For each co-morbidity, a list of diagnostic codes from hospital (ICD-10) and primary care coding schemes was used to identify diagnoses. The codes were compiled from online code repositories, including Cardiovascular Disease Research Using Linked Bespoke Studies and Electronic Health Records (CALIBER) [1] and medical dictionary keyword searches. These comorbidities were selected based on 3 sources: 1) the Quality and Outcomes Framework, a performance management and incentive scheme for general practitioners in the UK [2], 2) the Charlson Comorbidity Index, a commonly used co-morbidity index [3] and, 3) the US Department of Health and Human Services [4].

References

1. Denaxas SC, George J, Herrett E, Shah AD, Kalra D, Hingorani AD, et al. Data resource profile: cardiovascular disease research using linked bespoke studies and electronic health records (CALIBER). Int J Epidemiol. 2012;41(6):1625-38. Epub 2012/12/12. doi: 10.1093/ije/dys188. PubMed PMID: 23220717; PubMed Central PMCID: PMCPMC3535749.
2. NHS Digital. Quality and Outcomes Framework: NHS Digital; [26 Jul 2020]. Available from: https://qof.digital.nhs.uk/.
3. Charlson ME, Pompei P, Ales KL, MacKenzie CR. A new method of classifying prognostic comorbidity in longitudinal studies: development and validation. J Chronic Dis. 1987;40(5):373-83. Epub 1987/01/01. doi: 10.1016/0021-9681(87)90171-8. PubMed PMID: 3558716.
4. US Department of Health and Human Services Office of the Assistant Secretary for Health. HHS Initiative on Multiple Chronic Conditions 2015 [26 Jul 2020]. Available from: https://www.hhs.gov/ash/about-ash/multiple-chronic-conditions/index.html.
