# Supplementary material for: Multi-morbidity and blood pressure trajectories in hypertensive patients: A multiple landmark cohort study
Source: PLoS Med. 2021 Jun 17;18(6):e1003674. doi: 10.1371/journal.pmed.1003674 (PMC8248714; doi:10.1371/journal.pmed.1003674)
Supplement: S3 Table — (PDF) [file pmed.1003674.s011.pdf]

S3 Table. Anti-hypertensive British National Formulary codes and drug classes.

Codes	Description	Class				
		A	B	C	D	O
2020100	Thiazides and related diuretics				✓	
2020200	Loop diuretics				✓	
2020300	Potassium sparing diuretics & aldosterone antagonists				✓	
2020400	Potassium sparing diuretics & compounds				✓	
2020800	Diuretics with potassium				✓	
2040000	Beta-adrenoceptor blocking drugs		✓			
2040100	Beta-adrenoceptor blocking drugs with diuretic		✓		✓	
2050000	Hypertension and heart failure		✓			
2050100	Vasodilator antihypertensive drugs					✓
2050200	Centrally-acting antihypertensive drugs					✓
2050300	Adrenergic neurone blocking drugs					✓
2050400	Alpha-adrenoceptor blocking drugs					✓
2050500	Renin-angiotensin system drugs					
2050501	Angiotensin-converting enzyme inhibitors	✓				
2050502	Angiotensin-ii receptor antagonists	✓				
2050503	Renin inhibitors					
2050504	Angiotensin-ii receptor antagonists with diuretic	✓			✓	
2050800	Other adrenergic neurone blocking drugs					✓
2060200	Calcium-channel blockers			✓		
2050500*	Renin-angiotensin system drugs					

Classes were defined as: A) Angiotensin-converting enzyme inhibitors and angiotensin II receptor blockers, B) beta-blockers, C) calcium-channel blockers, D) diuretics, O) other (including alpha-blockers, vasodilators, centrally-acting anti-hypertensives); *Each drug under this code was individually categorised into a class.
