# Supplementary material for: Multi-morbidity and blood pressure trajectories in hypertensive patients: A multiple landmark cohort study
Source: PLoS Med. 2021 Jun 17;18(6):e1003674. doi: 10.1371/journal.pmed.1003674 (PMC8248714; doi:10.1371/journal.pmed.1003674)
Supplement: S4 Table — (PDF) [file pmed.1003674.s012.pdf]

S4 Table. Descriptive statistics of blood pressure management before and after diagnosis of hypertension.

	Mean number of BP readings (SD)*		Mean number of antihypertensive classes prescribed (SD)#	
**Number of co-morbidities**	**Before diagnosis**	**After diagnosis**	**Before diagnosis**	**After diagnosis**
**Total**	8.0 (6.6)	15.5 (10.0)	0.3 (0.7)	1.5 (0.9)
**0**	6.3 (5.4)	15.1 (9.7)	0.1 (0.5)	1.5 (0.9)
**1**	7.7 (6.1)	15.6 (10.0)	0.2 (0.6)	1.5 (0.8)
**2**	9.0 (6.8)	15.7 (10.2)	0.3 (0.7)	1.5 (0.9)
**3**	10.2 (7.4)	15.9 (10.6)	0.5 (0.8)	1.6 (0.9)
**4**	11.3 (7.9)	16.0 (11.0)	0.6 (0.9)	1.7 (0.9)
**5+**	13.7 (9.0)	16.0 (11.8)	1.0 (1.1)	1.9 (1.0)

*Capture period was 5 years from diagnosis of hypertension, i.e., mean number of blood pressure (BP) readings were counted from 5 years before diagnosis to time of diagnosis, and from time of diagnosis to 5 years after diagnosis. #Capture period was 1 year from time of diagnosis of hypertension, i.e., mean number of anti-hypertensive classes prescribed were counted from 1 year before diagnosis to time of diagnosis, and from time of diagnosis to 1 year after diagnosis. All differences between groups statistically significant ($p<0.01$) using analysis of variance.
