# Supplementary material for: Multi-morbidity and blood pressure trajectories in hypertensive patients: A multiple landmark cohort study
Source: PLoS Med. 2021 Jun 17;18(6):e1003674. doi: 10.1371/journal.pmed.1003674 (PMC8248714; doi:10.1371/journal.pmed.1003674)
Supplement: S5 Table — (PDF) [file pmed.1003674.s013.pdf]

S5 Table. Patient characteristics in landmark cohorts of incident hypertensive patients between 2000 and 2014 in the UK.

Characteristic	Time after diagnosis of hypertension (years)					
	-5 (n=145,964)	-1 (n=273,493)	0 (n=295,487)	1 (n=260,823)	5 (n=158,573)	10 (n=52,393)
**Age [years], mean (SD)**	56.6 (12.7)	60.5 (13.1)	61.5 (13.1)	62.6 (12.9)	66.2 (12.4)	70 (11.5)
**<65, % (n)**	74.4 (108,551)	63.7 (174,146)	60.7 (179,239)	57.8 (150,784)	47.6 (75,421)	35.2 (18,424)
**≥65, % (n)**	25.6 (37,413)	36.3 (99,347)	39.3 (116,248)	42.2 (110,039)	52.4 (83,152)	64.8 (33,969)
**Women, % (n)**	49.0 (71,482)	50.3 (137,700)	50.7 (149,787)	51.0 (133,098)	51.9 (82,375)	53.9 (28,247)
**Number of comorbidities**						
**0**	47.9 (69,856)	39.3 (107,551)	36.1 (106,801)	27.6 (72,090)	17.6 (27,979)	10.7 (5,594)
**1**	29.3 (42,719)	29.5 (80,549)	29.3 (86,691)	29.7 (77,399)	25.2 (39,945)	19.3 (10,107)
**2**	14.0 (20,397)	16.8 (46,059)	17.7 (52,432)	20.6 (53,744)	22.1 (35,100)	21.1 (11,066)
**3**	5.7 (8,272)	8.2 (22,365)	9.2 (27,185)	11.6 (30,358)	15.5 (24,653)	17.6 (9,195)
**4**	2.1 (3,119)	3.7 (10,090)	4.3 (12,824)	5.9 (15,287)	9.4 (14,901)	12.7 (6,663)
**≥5**	1.1 (1,601)	2.5 (6,879)	3.2 (9,554)	4.6 (11,945)	10.1 (15,995)	18.6 (9,768)
**No. with blood pressure measurements, % with missing data [n]**	36,350<br>87.7 [259,137]	69,622<br>76.4 [225,865]	271,002<br>8.3 [24,485]	142,778<br>46.9 [126,051]	80,446<br>52.0 [87,144]	28,049<br>51.7 [30,052]

Each timepoint represents a landmark year cohort (see Methods).
