# Supplementary material for: Multi-morbidity and blood pressure trajectories in hypertensive patients: A multiple landmark cohort study
Source: PLoS Med. 2021 Jun 17;18(6):e1003674. doi: 10.1371/journal.pmed.1003674 (PMC8248714; doi:10.1371/journal.pmed.1003674)
Supplement: S6 Table — (PDF) [file pmed.1003674.s014.pdf]

S6 Table. Baseline characteristics by cardiometabolic co-morbidity.

Characteristic	All cases (n=295,487)	Cardiometabolic comorbidities	
		Yes (n=112,656)	No (n=182,831)
**Age [years], mean (SD)**	61.5 (13.1)	63.5 (13.0)	60.3 (13.0)
**<65, % (n)**	60.7 (179,239)	54.0 (60,780)	64.8 (118,459)
**≥65, % (n)**	39.3 (116,248)	46.0 (51,876)	35.2 (64,372)
**Women, % (n)**	50.7 (149,787)	48.3 (54,437)	52.2 (95,350)
**White ethnicity, % (n)**	96.1 (116,574)	95.8 (47,723)	96.3 (68,851)
	[174,197]	[62,848]	[111,349]
**Fifths of deprivation index, % (n)**			
**Q1 (least deprived)**	23.3 (68,902)	21.3 (23,961)	24.6 (44,941)
**Q2**	22.6 (66,699)	21.4 (24,080)	23.3 (42,619)
**Q3**	21.0 (62,142)	21.0 (23,663)	21.0 (38,479)
**Q4**	18.2 (53,722)	19.2 (21,610)	17.6 (32,112)
**Q5 (most deprived)**	14.8 (43,718)	17.1 (19,220)	13.4 (24,498)
**Mean SBP (SD), mmHg**	159.1 (21.1)	155.8 (20.7)	161.1 (21.1)
**Mean DBP (SD), mmHg**	91.0 (12.5)	88.0 (12.5)	92.9 (12.1)
**Body mass index, % (n)**			
**Underweight (<18.5 kg/m^2)**	0.9 (1,552)	0.7 (581)	1.0 (971)
**Normal (18.5 to 24.9 kg/m^2)**	22.0 (37,849)	18.1 (14,156)	25.2 (23,693)
**Overweight (25 to 29.9 kg/m^2)**	38.6 (66,484)	35.1 (27,482)	41.5 (39,002)
**Obese (≥30 kg/m^2)**	38.5 (66,403)	46.1 (36,117)	32.2 (30,286)
	[123,199]	[34,320]	[88,879]
**Smoking status, % (n)**			
**Current**	20.1 (44,376)	18.9 (16,991)	21.0 (27,385)
**Never**	47.7 (105,304)	44.7 (40,195)	49.9 (65,109)
**Ex-smoker**	32.1 (70,871)	36.5 (32,809)	29.2 (38,062)
	[74,936]	[22,661]	[52,275]
**Mean total cholesterol (SD), mmol/L**	5.5 (1.2)	5.3 (1.3)	5.7 (1.0)
	[104,581]	[23,989]	[80,592]
**Year of hypertension diagnosis, % (n)**			
**2000**	5.0 (14,822)	4.4 (4,918)	5.4 (9.904)
**2014**	3.4 (10,129)	4.0 (4,477)	3.1 (5,652)
**Number of anti-hypertensive classes prescribed, % (n)**			
**1**	13.9 (41,137)	18.7 (21,113)	11.0 (20,024)
**2**	6.5 (19,267)	10.4 (11,670)	4.2 (7,597)
**≥3**	2.7 (8,058)	5.0 (5,620)	1.3 (2,438)

The category percentages refer to complete cases. Numbers in square brackets are numbers with missing data for the relevant characteristic. Deprivation level refers to Index of Multiple Deprivation 2015, where Q1 is least deprived fifth and Q5 is most deprived fifth of the population. SBP – systolic blood pressure; DBP – diastolic blood pressure. Number of anti-hypertensive classes refers to anti-hypertensives grouped into 5 classes: angiotensin-converting enzyme inhibitor and angiotensin II receptor blocker, beta-blockers, calcium channel blockers, diuretics, and other (see Methods and S3 Table).
