# Supplementary material for: Multi-morbidity and blood pressure trajectories in hypertensive patients: A multiple landmark cohort study
Source: PLoS Med. 2021 Jun 17;18(6):e1003674. doi: 10.1371/journal.pmed.1003674 (PMC8248714; doi:10.1371/journal.pmed.1003674)
Supplement: Attachment — Submitted filename: Response_Feedback_Final.docx [file pmed.1003674.s015.docx]

Dear Dr Misra,

Many thanks for the feedback from your editorial team and those from the reviewers, which we find very insightful. We have considered the various suggestions and clarified aspects in the manuscript that required further explanation or additional information. We also made changes in the manuscript to improve clarity and consistency in the terms that we used. We provide a version where we show changes that have been made (page and line locations are based on this copy of the manuscript), as well as a clean copy of this revision. In addition, we noted an error in the list of authors added into the system. We have missed adding Dr D Canoy (whose name appears in the version originally submitted) which we now rectify.

We appreciate giving us the opportunity to revise and improve our manuscript, and we hope that the changes we have made are sufficient to address queries from the editorial team and the reviewers. Do let us know should you have any further query.

Kind regards,

Kazem Rahimi

-----------------------------------------------------------------------------------------------------------

Requests from the editors:

**Title**

**Please revise your title according to PLOS Medicine's style. Your title must be nondeclarative and not a question. It should begin with main concept if possible. "Effect of" should be used only if causality can be inferred, i.e., for an RCT. Please place the study design ("A randomized controlled trial," "A retrospective study," "A modelling study," etc.) in the subtitle (ie, after a colon).**

Response:

We have now modified the title to ‘Multimorbidity and blood pressure trajectories in hypertensive patients: a multiple landmark cohort study’ (title page).

**Competing interests**

**Please add this statement to the manuscript's Competing Interests: "[Initials] is an Academic Editor on PLOS Medicine's editorial board."**

Response: We have now added this competing interest statement at the end of the manuscript.

**DAS**

**The Data Availability Statement (DAS) requires revision. For each data source used in your study:**

**a) If the data are freely or publicly available, note this and state the location of the data: within the paper, in Supporting Information files, or in a public repository (include the DOI or accession number).**

**b) If the data are owned by a third party but freely available upon request, please note this and state the owner of the data set and contact information for data requests (web or email address). Note that a study author cannot be the contact person for the data.**

**c) If the data are not freely available, please describe briefly the ethical, legal, or contractual restriction that prevents you from sharing it. Please also include an appropriate contact (web or email address) for inquiries (again, this cannot be a study author).**

Response:

We have now added a statement on availability of study data at the end of the manuscript.

**Abstract**

*** Please structure your abstract using the PLOS Medicine headings (Background, Methods and Findings, Conclusions).**

*** Please combine the Methods and Findings sections into one section, “Methods and findings”.**

*** Please ensure that all numbers presented in the abstract are present and identical to numbers presented in the main manuscript text. * Please include the study design, population and setting, number of participants, years during which the study took place, length of follow up, and main outcome measures. * Please quantify the main results (with 95% CIs and p values). * Please include the important dependent variables that are adjusted for in the analyses.**

Response:

We have modified the abstract as suggested, and included estimates with their 95% CI. Given the sample size and tight confidence intervals, we do not think the P values will add any additional information hence we did not include these data.

**Conclusions**

*** Please address the study implications without overreaching what can be concluded from the data; the phrase "In this study, we observed ..." may be useful. * Please interpret the study based on the results presented in the abstract, emphasizing what is new without overstating your conclusions. * Please avoid vague statements such as "these results have major implications for policy/clinical care". Mention only specific implications substantiated by the results.**

**Please revise “multimorbid patients” to “patients with multi-morbidities” or similar**

Response:

We have now modified this section of the abstract.

**We do not require a key messages section. We ask that you include a short, non-technical Author Summary of your research to make findings accessible to a wide audience that includes both scientists and non-scientists. The Author Summary should immediately follow the Abstract in your revised manuscript. This text is subject to editorial change and should be distinct from the scientific abstract. Please see our author guidelines for more information: https://journals.plos.org/plosmedicine/s/revising-your-manuscript#loc-author-summary**

**Please place reference square brackets after punctuation**

**Please revise comorbidities throughout to “co-morbidities”**

Response:

We have now deleted this section, and made changes to the in-text citation format and use of the terminologies ‘multi-morbidity’ and ‘co-morbidity’ in the manuscript. We have also added the ‘Author Summary’ section (page 5).

**Introduction**

**The first few sentences are identical to the start of the abstract, please revise**

Response:

We have now altered this section in the introduction.

**Methods**

**Please briefly describe the landmark cohort design**

Response:

We have provided additional detail about this cohort design (page 8, line 133-139).

**Please ensure that the study is reported according to the [STROBE] guideline, and include the completed [STROBE or other] checklist as Supporting Information. When completing the checklist, please use section and paragraph numbers, rather than page numbers. Please add the following statement, or similar, to the Methods: "This study is reported as per the Strengthening the Reporting of Observational Studies in Epidemiology (STROBE) guideline (S1 Checklist)."**

Response:

The STROBE checklist is now included as an additional file.

**Did your study have a prospective protocol or analysis plan? Please state this (either way) early in the Methods section.**

**a) If a prospective analysis plan (from your funding proposal, IRB or other ethics committee submission, study protocol, or other planning document written before analyzing the data) was used in designing the study, please include the relevant prospectively written document with your revised manuscript as a Supporting Information file to be published alongside your study, and cite it in the Methods section. A legend for this file should be included at the end of your manuscript.**

**b) If no such document exists, please make sure that the Methods section transparently describes when analyses were planned, and when/why any data-driven changes to analyses took place.**

**c) In either case, changes in the analysis-- including those made in response to peer review comments-- should be identified as such in the Methods section of the paper, with rationale.**

Response:

We have indicated in the Method section that the research protocol was approved by the CPRD ISAC committee (page 8, line 128-131). The analyses required to address queries of the reviewers will not require modification of the protocol research plan.

**Results**

**Please provide exact p-values along with 95%CI**

Response:

We do not think it is necessary to provide P values where we report adjusted mean values of SBP and their corresponding 95% CI. Given the size of the study population and the tight confidence intervals, the P values do not add any additional statistical meaning to the results being presented.

**Please simplify this sentence “Patterns were similar in years before and at hypertension diagnosis across diseases, but with larger differences in years before hypertension diagnosis than years after diagnosis (Figure S3)”**

Response:

We revised this statement and it now reads as “The pattern of lower SBP in patients with cardiometabolic co-morbidities, when compared to those without such co-morbidities, was also seen when we retrospectively compared SBP in the years before diagnosis of hypertension. This difference was also larger in magnitude in years prior to, rather than at the time of, the diagnosis of hypertension (Figure S3).” (page 15, line 288-292)

**Please revise Antihypertensive to “Anti-hypertensive”**

Response:

We have made changes in the use of this term in the manuscript.

**Discussion**

**Please simplify this sentence “we could identify and quantify the impact of additional reasons for the lower BP in people with more comorbidities”**

Response:

We now modified this clause to “….we could identify some factors that might explain the lower BP observed in people with more co-morbidities and provide quantitative estimates of their impact on BP ” (page 18, line 354-356).

**“such as ischemic heart disease, atrial fibrillation and diabetes, lower BP was partly driven by a higher rate of antihypertensive use. However, BP levels were also lower in patients” please replace driven by to “associated with” to avoid overstating findings from an observational study**

Response:

We have modified this to “More specifically, we found that in patients with pre-existing cardiometabolic co-morbidities, such as ischaemic heart disease, atrial fibrillation and diabetes, their lower BP was partly explained by a higher proportion of anti-hypertensive medication use. Adjustment for this treatment slightly attenuated the difference in SBP between those with and without cardiometabolic co-morbidity but the BP levels remained lower in patients with other co-morbidities despite similar intensity of anti-hypertensive treatment.” (page 18, 358-364).

**Please rephrase this for clarity and accuracy “By tracking patients forwards and backwards in time”**

Response:

We have now modified this phrase to “By retrospectively and prospectively determining SBP levels from the time of the diagnosis of hypertension….” (page 19, line 366-369).

**Bibliography- please use Vancouver style**

Response:

The bibliography has been modified according to Vancouver style.

Comments from the reviewers:

**Peter Flom**

**Reviewer #1: I confine my remarks to statistical aspects of this paper. These were mostly fine, but I have a couple minor issue to resolve before I can recommend publication.**

**NOTE: Line numbers would have made the review easier**

Response:

Many thanks for the suggestion. We have now added line numbers to the manuscript.

**p. 6 Describe "quality data" at least a little. Don't just cite a paper.**

Response:

This is a term that CPRD uses to classify the data that they share. The CPRD monitors and provides information about the data meeting research quality standards at practice and patient levels. We revised and added some text to describe the quality of the data resource (Page 8, 1^st^ paragraph).

**p 7 Please descibe what a "multiple landmark cohort design" is.**

Response:

We have now briefly expanded the description of this study design (page 8, last paragraph).

**p. 8 Give more detail about how the values were used to get mean SBP**

Response:

This is now explained in fuller detail on page 9, last paragraph.

**Don't categorize BMI. Categorizing any continuous IV is almost always a mistake., In *Regression Modelling Strategies* Frank Harrell lists 11 problems with this. For BMI in particular, see my post https://medium.com/peter-flom-the-blog/why-bmi-is-a-bad-measure-of-obesity-and-what-is-better-f8a62fc9ca49**

Response:

Many thanks for pointing this out. We agree that using BMI as a continuous variable might result in a better model fit for the data than when using it as a categorical variable. Indeed, some of the authors also have substantive experience in handling anthropometric measures as well as other phenotypic indicators of body size and shapes in large population studies and are aware of this issue. Despite this better model fit, we also expect that the estimates of the outcome are likely to remain similar when using continuous and categorical forms of BMI as a covariate in the model in this large-scale study. We re-analysed our data using BMI as a continuous variable in our models, and as expected, the results, e.g., as shown in Figure 1, remain similar to our previous findings. Nevertheless, we report these updated results. In addition, we report mean BMI, rather than proportions according to BMI categories, in Table 1. Alternative indicators of excess adiposity are not recorded in routine primary care health records in the UK so we could not use measurements other than BMI.

**p. 11 These effects are pretty small. Are they clinically important? I actually take my BP daily and it varies by more then 5 points from day to day**

Response:

We agree that BP is variable and can fluctuate widely within individuals. However, our estimates in this large-scale population study are based on group averages with very narrow confidence intervals. A 5-mmHg difference in SBP as found in our current analysis is clinically meaningful at a population level. This for instance is evidenced by our recent presentation at the European Society of Cardiology Annual Congress summarising the findings of 51 randomized clinical trials (350,000 participants) of pharmacologic blood pressure-lowering treatment. This found that on average, a 5-mmHg reduction in SBP leads to a reduction in cardiovascular disease risk by 10%. A 5 mmHg difference (controlled for within and between-person variabliy) is roughly equivalent to use of one antihypertensive drug.

**Reviewer #2: Men and women have very different distributions of blood pressure and comorbidities. Also, there is a large difference between men and women in blood pressure changes after age 60s. Thus sex-stratified analysis should be presented as the main analysis, not as a sensitivity analysis.**

Response:

Many thanks for the suggestion. We have now redone sex-stratified analysis for our main analysis, which show that the patterns pertaining to our research objective (relationship between comorbidity status and BP repeated over time) are similar to those observed for the full cohort. We have therefore kept the summary figure in the main report but now present the additional figures for the mean SBP over time by number of co-morbidities (Figure S4 and S5), and the mean differences in SBP at one year after hypertension diagnosis stratified by co-morbidity status (Figure S6 and S7) in the supplement.

**It seems that people who took anti-hypertensive drugs before and after the diagnosis of hypertension were included. Also, the prescription before and after diagnosis is not clearly distinguished. People with other diseases would be more likely to take anti-hypertensive drugs before hypertension diagnosis. It can affect the mean blood pressure levels of different groups. If authors want to see the effects of comorbidities on blood pressure level, people with a history of taking anti-hypertensive drugs should be excluded and analyzed.**

Response:

We fully agree that antihypertensive use can affect mean blood pressure level (a finding that is indeed supported by our own analysis (page 16, line 303 onwards). The underlying motivation for this work was to understand why some of the evidence in the literature suggest that patients with multi-morbidity tend to have lower BP, or have downward BP trajectory over time. It is unclear if these observations are likely to be due to biological reasons (as argued in previous work) or whether there are alternative explanations, such as biases or confounding factors, that were not accounted for. One such factor we considered was the use of anti-hypertensive treatment. We added some information on potential variation on BP management, as indicated by frequency of BP readings and number of anti-hypertensive drug classes prescribed (Table S4), which seem to suggest that there is more intense monitoring and treatment in hypertensive patients with more co-morbidities. We have adjusted for antihypertensive treatment and showed some attenuation in the SBP difference between those with less and more co-morbidities (Figure 3). Nevertheless, the SBP difference according to co-morbidity status persisted. An alternative approach to take account for the impact of anti-hypertensive use is to exclude those prescribed with this medication. However, this approach will exclude a substantial proportion of people (hypertension is highly prevalent, and use of anti-hypertensive drugs for its treatment and other indications is common), which would lead to findings with limited generalisability.

**Many of the comorbidites, which were included in the study, are also associated with high blood pressure or its risk factors. Such hypertension-related diseases and other diseases should be analyzed separately.**

Response:

We have reported differences in SBP by type of comorbidities (Fig 2). As expected, the largest differences were seen in patients with cardiometabolic co-morbidities. However, we still see these differences, albeit at smaller magnitudes, with co-morbidities that were not related to cardiometabolic conditions. Indeed, this finding emerged precisely because we did not a priori exclude patients with a particular comorbidity (as done in some previous reports). Rather, we describe the patterns in the overall incident hypertension population, and then investigate the effects of various factors affecting blood pressure (and track changes over time).

**Although the authors have mentioned implications for future research and practice, I am not exactly aware of the clinical significance of the results from this study. Please make clear what this study is trying to evaluate: the effects of comorbidities on blood pressure levels; the effects of comorbidities on the diagnosis of hypertension; or the effects of comorbidities on the management of diagnosed hypertension. Depending on the purpose of the study, the selection criteria and analysis methods may vary.**

Response:

We aimed to investigate the burden and types of co-morbidities in patients with hypertension (key population selection criterion), and to assess how such co-morbidities and other variables (such as treatment and physician interaction as exposure variables) affect blood pressure levels (our outcome variable) over time (using a multiple landmark design to take account of changing comorbidity and treatment status). This in essence is an extended version of the first option that the reviewer is offering. The purpose was to find an explanation why hypertensive patients with multi-morbidity tend to have lower BP than those without, as reported in other studies. These earlier observations have been interpreted to mean that patients with multi-morbidity will not require BP-lowering treatment because of their lower BP, affecting international guidelines. However, our findings suggest that there is already disparity in BP levels according to number or type of co-morbidity even at around the time of the diagnosis of hypertension. Hence, our study suggests that ignoring this difference could lead to inappropriate conclusions (ie, the disparity by multi-morbidity status being causal). This difference at some ‘baseline’ time point needs to be considered when assessing and monitoring subsequent BP in patients with multi-morbidity, otherwise clinicians might unwittingly withhold treatment because of perceived notion of declining SBP trajectory in patients with multi-morbidity. We have now revised the manuscript throughout for clarity of the objectives and implications.

**Reviewer #3: This paper described the association between SBP and comorbidities in participants with hypertension. It is interesting to provide evidence for guiding antihypertension therapy. However, there are some major concerns as explained below. I hope the following comments would be useful for the authors to improve this study.**

**1. As a real-work study based on the linked electronic health records, I think it's important to provide a flowchart to describe how the authors selected the patients from the study population. This will facilitate readers to interpret the results for the generalization and judge any selection bias existed in the study population.**

Response:

We have now added this flowchart in the supplement (Figure S2).

2. **It will be very helpful to provide the number of patients without imputation to actually have BP measurement at each year before or after the hypertension diagnosis. It's suspected to see that even five years before the new diagnosis of hypertension, the mean BP levels in all these patients were above 140. It may be argued that there was missing not at random for this type of data, i.e. their BP may be recorded only when its value is above 140.**

Response:

In supplementary Table S5, we added information on actual numbers with and without BP measurements at selected landmark years before and after the diagnosis of hypertension. Please note that we do not dispute that recording of blood pressure is likely to be biased. Indeed, this is the key conclusion from our study: we show that observations from earlier studies that blood pressure ‘declines’ faster in people with multimorbidity is likely to be an artefact of several recording (and treatment) biases. Most importantly, people with differing disease status seems to be diagnosed with hypertension (which is dichotomisation of a continuous variable) at different thresholds. This ‘diagnosis bias’ was found to the most important determinant of BP changes over time.

**3. How did the authors accommodate the within-subject correlations when assessing the association between SBP and comorbidities?**

Response:

We imputed SBP values for landmark time points for which patients did not have recorded SBP (up until they ‘exit’ or ‘censored’ from the analysis). This imputation takes into account ‘nearby’ values. Thus, the repeated SBP for an individual are expected to be correlated particularly in this scenario. However, in this landmark design, patients may be reclassified, for example, when they gain another co-morbidity in another landmark timepoint. Thus, some patients may contribute data to a different co-morbidity category across the landmark years. Then, we ran separate multivariable models for each landmark year to estimate the BP by co-morbidity status for that landmark cohort. Since different patients may contribute to different co-morbidity category at different landmark time points, and separate multivariable analyses were conducted for each of these time points, within-subject correlation will be less of an issue.

**4. The authors stated that newly diagnosed hypertension are likely to accrue additional comorbidities referred in the Key messages. How does the results support this? This study population was old (mean age, 61.6y) and it's not clear the evolution of comorbidity in participants without diagnosed hypertension. Patients without any additional comorbidity were still majority of the population.**

Response:

Many thanks for pointing this out. Indeed, the mean age is nearly 62 years, but about half of the study population were below this age (criterion for minimum age was ≥16 years), so there are substantial numbers of patients who were relatively in the younger adult age group. In the results section (page 14, line 256-261), we referred to the proportions of those without co-morbidities and those with ≥5 comorbidities at baseline (year of hypertension diagnosis) and at 10-year landmark timepoint. The proportions of those without comorbidity at the time of the diagnosis and at 10-year landmark time point were 36.1% and 10.7%, respectively; in contrast, the proportions of those with ≥5 comorbidities at the same respective time points were 3.2% and 18.6%, respectively. Thus, over time, the proportions of those accruing additional comorbidity have increased. Details of these results are also shown in Table S5.

**5. Where did the authors extract the BP measurements from the EHRs? Will it be different for patients with or without comorbidities? For example, patients without comorbidities may be measured by GP whereas patients with other comorbidities could be obtained from the in-patient data.**

Response:

All data were extracted from records of blood pressure during visits to the general practice clinics. We have not used any in-patient BP data in our analyses.

**6. It is not clear how to calculate SBP as time-updated covariates. If it was stated in Text S1, please quote this appendix in the Method.**

Response:

Data capture for SBP was conducted for each landmark year, using recorded SBP within 3 months of a landmark timepoint (illustrated in orange colour in Figure S1) after assessing exposure status (ie, co-morbidity status). Over time, some patients accrue additional co-morbidities, thus, changing their exposure status in another landmark year. Thus, the exposure, outcome and any potential confounders will be updated at each landmark year. We then explored the relationship between co-morbidity status and SBP by running multivariable models separately for each landmark year. We provided additional description of this data capture in the text (page 9, line 154-163). We have omitted Text S1.

**7. As mentioned in the limitations, missing data was inevitable in EHR data. It's better to briefly report the missing rate of covariates and missing pattern if rate is high.**

Response:

The numbers with missing data for the covariates are shown in Table 1 (numbers shown in square brackets). We have now added in the text (page 13, lines 236-238) the description of the total study population including information on the proportions with missing values for the covariates. Please note that data for certain variables are complete or assumed to be complete. For instance, deprivation level is complete because this information is derived by the data provider using area-based information of each individual. The nature of the linked dataset used also justifies assuming that diagnosis and medication recording are complete, as in previous epidemiological studies. Limitations relating to data missingness are discussed (page 20, line 403-419).
