# Supplementary material for: Multi-morbidity and blood pressure trajectories in hypertensive patients: A multiple landmark cohort study
Source: PLoS Med. 2021 Jun 17;18(6):e1003674. doi: 10.1371/journal.pmed.1003674 (PMC8248714; doi:10.1371/journal.pmed.1003674)
Supplement: Attachment — Submitted filename: Response_revision2_Final.docx [file pmed.1003674.s016.docx]

**Comments from the reviewers:**

**Reviewer #1:** The authors have addressed my concerns and I now recommend publication.

**Response:** Thank you.

**Reviewer #3:** This paper described the association between SBP and comorbidities in participants with hypertension. Though the dataset is big which described the association between SBP and comorbidities to guide anti-hypertension therapy, there are some major concerns as explained below:

1. In this revision, the authors added information on actual numbers with and without BP measurements and stated that recording of blood pressure is likely to be biased. Considering the assumption of imputation method (mostly missing at random) required, I'm not convinced that this way to deal with missing data is valid. Furthermore, the conclusion based on this, in my opinion, is not solid. The authors claimed, in fact, they would like to conclude that the association is caused by the recording (and treatment) and diagnosis biases. Then this seems to be a false association between SBP and comorbidities to me. Moreover, how could this conclusion help to guide the clinical practice?

**Response:** Thank you for raising these important points, which have given us another impetus to clarify our approach and conclusions of the paper. We explain the purpose and technique of imputation relating to BP in our methods (**lines 158-165**).

The purpose of this was to leverage the temporal correlation of repeated BP measurements within individuals to predict the missing values at a specific landmark timepoint. In **Table S4**, we further report the average number of BP readings for patients stratified by number of comorbidities at baseline. As the table shows, on average there were 8 repeated measures per individual in the 5 years prior to hypertension diagnosis and 15.5 in the 10 years after diagnosis. As the table also shows and as one would expect in routine clinical care, people with more comorbidities were more likely to have their BP measured (with a greater difference prior to diagnosis of hypertension than after diagnosis). This differential recording pattern refers to an indication of informed presence bias (a type of measurement bias). We further show in a sensitivity analysis that adjustment for the frequency of recording attenuates the difference in SBP between patient groups but does not explain the pattern fully. The implications of this are discussed (**lines 367 to 385**). Additionally, we perform a sensitivity analysis that restricts analysis to complete cases and find no material difference in outcomes (methods **lines 209-211**, and discussion **lines 334-336**). In our discussion, we further refer to the limitation of missing information and cite supporting evidence from the Framingham study, which while not subject to the same biases, observed a similar rising pattern in BP before hypertension diagnosis (**lines 416-423**)

In terms of interpretation of the patterns observed, we agree that one should not conclude that it is the burden or type of comorbidity that drives the differences in BP between groups of individuals. This is supported by a range of adjustments, and importantly by our temporal analysis which reveals that there is no faster decline in BP in people with more multimorbidity. So far, major clinical practice guidelines for BP management (NICE, ESC and AHA, ACP/AAPF) have interpreted the limited and largely cross-sectional evidence on lower BP in people with multimorbidity as causal to conclude that patients with multi-morbidity do not require intensive BP-lowering treatment because of their lower BP. However, our findings suggest that there is already a disparity in BP levels according to the number or type of co-morbidity, even at the time of the diagnosis of hypertension. Hence, our study suggests that ignoring this difference could lead to inappropriate conclusions. This difference at some ‘baseline’ time point needs to be considered when assessing and monitoring subsequent BP in patients with multi-morbidity, otherwise clinicians might unwittingly withhold treatment because of the perceived notion of declining SBP trajectory in patients with multi-morbidity. The lack of a more rapid decline in SBP in those with multi-morbidity provides some reassurance for BP treatment in these high-risk individuals. We have now revised the manuscript including the abstract conclusion to clarify this implication.

2. The authors stated that "newly diagnosed hypertension are likely to accrue additional comorbidities" in the first sentence of the discussion. They draw this conclusion based on the observation that the proportion of those with ≥5 comorbidities increased at 10-year landmark time point. I don't really agree with this because this could be just age effect. The comparison between patients with and without hypertension is the direct support to this statement where the current study has only patients with hypertension.

**Response:** Please note that we do not intend to imply that hypertension causes more comorbidities than what would be expected when an individual has not been diagnosed with hypertension (in which case, as the reviewer suggests, a comparison of the findings with those without hypertension would be necessary). Rather, this statement merely summarises the observation made in our study which was confined to people with hypertension only. Thus, we specify in which population that observation was made to avoid any overgeneralisation. We have now reworded the sentence which now reads:

*“In this large-scale cohort study of patients with newly diagnosed hypertension, we observed a pattern of accumulating co-morbidities over time.”*
