# Supplementary material for: Multi-morbidity and blood pressure trajectories in hypertensive patients: A multiple landmark cohort study
Source: PLoS Med. 2021 Jun 17;18(6):e1003674. doi: 10.1371/journal.pmed.1003674 (PMC8248714; doi:10.1371/journal.pmed.1003674)
Supplement: Attachment — Submitted filename: Response_to_editor_comments.docx [file pmed.1003674.s017.docx]

Dear Dr Turner:

Many thanks for your comments to our manuscript. We have now modified and revised the manuscript to take into account the suggestions and comments that have been made. Our specific responses and changes made are detailed herein.

Kind regards,

Kazem Rahimi

-----------------------------------------------------------------------------------------------

1. Please adapt the relevant sentence in the competing interest statement (submission form) to "KR is a member of PLOS Medicine's Editorial Board", or similar.

Re: We have now added the requested sentences.

2. At line 24 of your abstract, please quote aggregate demographic details for study participants.

Please also mention the country and data source.

Re: We have now added the requested information in abstract. This section of the abstract now reads:

“*In this multiple landmark cohort study, we used linked electronic health records from the UK Clinical Practice Research Datalink (CPRD) to compare systolic blood pressure (SBP) levels in 295,487 patients (51% women) aged 61.5 (SD=13.1) years with first recorded diagnosis of hypertension between 2000 and 2014,*”

3. Please add a new final sentence to the "Methods and findings" subsection of your abstract, which should begin "Study limitations include ..." or similar and should quote 2-3 of the study's main limitations.

Re: We have now added the requested the following text in the abstract:

“*Nevertheless, some limitations have to be considered including the possibility that diagnosis of some conditions may not have been recorded, varying degrees of missing data inherent in analytical datasets extracted from routine health records, and greater measurement errors in clinical measurements taken in routine practices than those taken in well-controlled clinical study settings.*”

4. You mention a research protocol at line 132. Please attach the relevant document, in addition to a prespecified analysis plan if available, as an attachment, referred to the in text. Please highlight analyses that were not prespecified.

This protocol refers to the scientific proposal that we have submitted to the CPRD ISAC. This protocol involved a proposed programme of research to which this current study is one of the proposed activities. Whilst the CPRD ISAC does not publish in full the protocols it approves, the summary of these protocols are shown on their website. The CPRD website link in the manuscript should be sufficient for readers to find where this summary might be found.

5. Please remove the information on competing interests, funding and data access from the end of the main text. This information will appear in the article metadata, via entries in the submission form.

Re: We have not removed this information, and will add these details instead in the submission form.

6. Please use the abbreviation "PLoS Med." consistently in your reference list.

Re: We have now altered the abbreviation as suggested.

7. Please move figure S2 to the main body of the paper.

Re: We have now moved Figure S2 as a Figure in the main text.

8. Please adapt the label of the attached STROBE checklist to "S1_STROBE_Checklist" or similar, and refer to the file by this label in your methods section.

Re: This supplementary file has now been renames as suggested.

9. At line 214 you refer to RECORD: did you mean to refer to STROBE? Please feel free to substitute a completed RECORD checklist if not.

Re: Many thanks for pointing out this error. We have updated this section in the text to refer to STROBE, and revised the relevant citation in the bibliography.

10. In the checklist, individual items should be referred to by section (e.g., "Methods") and paragraph number not by line or page numbers, as the latter change upon publication.

Re: We have renamed the column as ‘Section and location’, and updated the entries to refer to the relevant sections and paragraph number for each of the items in the checklist.
